# Orai1 Channel Regulates Human-Activated Pancreatic Stellate Cell Proliferation and TGF_β1_ Secretion through the AKT Signaling Pathway

**DOI:** 10.3390/cancers13102395

**Published:** 2021-05-15

**Authors:** Silviya Radoslavova, Antoine Folcher, Thibaut Lefebvre, Kateryna Kondratska, Stéphanie Guénin, Isabelle Dhennin-Duthille, Mathieu Gautier, Natalia Prevarskaya, Halima Ouadid-Ahidouch

**Affiliations:** 1 Laboratory of Cellular and Molecular Physiology, UR-UPJV 4667, University of Picardie Jules Verne, 80039 Amiens, France; silviya.radoslavova@etud.u-picardie.fr (S.R.); th.lefebvre27@gmail.com (T.L.); isabelle.dhennin@u-picardie.fr (I.D.-D.); mathieu.gautier@u-picardie.fr (M.G.); 2 University of Lille, Inserm U1003–PHYCEL–Cellular Physiology, 59000 Lille, France; antoine.folcher@inserm.fr (A.F.); kateryna.kondratska@inserm.fr (K.K.); natacha.prevarskaya@univ-lille.fr (N.P.); 3 Centre de Ressources Régionales en Biologie Moléculaire, UFR des Sciences, 80039 Amiens, France; stephanie.vandecasteele@u-picardie.fr

**Keywords:** activated pancreatic stellate cells, Orai1 channel, TGF_β1_, cell proliferation, AKT activation

## Abstract

**Simple Summary:**

Activated pancreatic stellate cells (aPSCs), the main source of cancer-associated fibroblasts in pancreatic ductal adenocarcinoma (PDAC), are well known as the key actor of the abundant fibrotic stroma development surrounding the tumor cells. In permanent communication with the tumor cells, they enhance PDAC early spreading and limit the drug delivery. However, the understanding of PSC activation mechanisms and the associated signaling pathways is still incomplete. In this study, we aimed to evaluate the role of Ca^2+^, and Orai1 Ca^2+^ channels, in two main PSC activation processes: cell proliferation and cytokine secretion. Indeed, Ca^2+^ is a versatile second messenger implicated in the regulation of numerous biological processes. We believe that a better comprehension of PSC Ca^2+^ -dependent activation mechanisms will bring up new crucial PDAC early prognostic markers or new targeting approaches in PDAC treatment.

**Abstract:**

Activated pancreatic stellate cells (aPSCs), the crucial mediator of pancreatic desmoplasia, are characterized, among others, by high proliferative potential and abundant transforming growth factor _β1_ (TGF_β1_) secretion. Over the past years, the involvement of Ca^2+^ channels in PSC pathophysiology has attracted great interest in pancreatic cancer research. We, thus, aimed to investigate the role of the Orai1 Ca^2+^ channel in these two PSC activation processes. Using the siRNA approach, we invalided Orai1 expression and assessed the channel functionality by Ca^2+^ imaging, the effect on aPSC proliferation, and TGF_β1_ secretion. We demonstrated the functional expression of the Orai1 channel in human aPSCs and its implication in the store-operated Ca^2+^ entry (SOCE). Orai1 silencing led to a decrease in aPSC proliferation, TGF_β1_ secretion, and AKT activation. Interestingly, TGF_β1_ induced a higher SOCE response by increasing Orai1 mRNAs and proteins and promoted both AKT phosphorylation and cell proliferation, abolished by Orai1 silencing. Together, our results highlight the role of Orai1-mediated Ca^2+^ entry in human aPSC pathophysiology by controlling cell proliferation and TGF_β1_ secretion through the AKT signaling pathway. Moreover, we showed a TGF_β1_-induced autocrine positive feedback loop by promoting the Orai1/AKT-dependent proliferation via the stimulation of Orai1 expression and function.

## 1. Introduction

Extensive desmoplastic stroma is the central pathological feature of pancreatic ductal adenocarcinoma (PDAC), responsible for the tumor’s development, progression, metastasis, and treatment resistance. This fibrotic stroma is mainly composed of cancer-associated fibroblasts (CAFs), also called pancreatic stellate cells (PSCs). Indeed, activated PSCs are the orchestrators of fibrotic desmoplasia development and the major source of CAFs in PDAC [1,2,3,4].

In the healthy pancreas, PSCs are stromal vitamin A lipid-storing cells residing in an inactive-quiescent state that accounts for 4–7% of the organ. Quiescent PSCs are known to maintain pancreatic tissue architecture by sustaining the balance between extracellular matrix (ECM) secretion and degradation. However, in response to pancreatic injury, inflammation, or carcinogenic processes, PSCs undergo morphological and functional modifications to acquire a myofibroblast-like phenotype and become activated. This transition is followed by a loss of vitamin A-containing lipid droplets, an increase in alpha-smooth muscle actin (αSMA) expression, and ECM secretion, such as type I collagen. PSC activation induces the enhancement of their proliferative and migratory potentials, leading to the development of the dense fibrotic tissue surrounding the pancreatic cancer cells (PCCs) and disrupting the drug delivery [5,6,7,8]. Indeed, during PDAC, this desmoplastic reaction formed by the PSC-induced fibrotic tissue accounts for 80% of the tumor’s total volume. In fact, it is well known that activated PSCs establish a dynamic dialogue with the PCCs, and profoundly affect the tumor cell behavior by promoting PCCs proliferation, migration, and invasion to enhance PDAC early spreading [4,9].

PSC activation is also characterized by abundant secretion of various cytokines, chemokines, and growth factors, such as the transforming growth factor _β1_ (TGF_β1_) [10,11]. TGF_β1_ is well known now as the profibrotic critical regulator of pancreatic fibrosis, which drives PSC activation through the regulation of αSMA expression, cell proliferation, and ECM synthesis, mainly by type I collagen synthesis [12,13,14]. This TGF_β1_-mediated autocrine loop contributes to the sustained activation of PSCs [15,16,17].

All these cellular processes implicated in PSC activation are controlled by intracellular signal transduction pathways [10,18,19]. Among these pathways, the serine-threonine kinase AKT has been reported to regulate PSC proliferation and cell cycle progression. Schwer et al. have demonstrated that blockade of the PI3K/AKT pathway with carbon monoxide releasing molecule-2 (CORM-2) inhibited PSC proliferation and induced the cell cycle arrest in the G0/G1 phase [20]. Moreover, Zhang et al. highlighted the effect of the tumor suppressor PTEN (phosphatase and tensin homolog), known to reduce AKT phosphorylation, on the inhibition of PSC proliferation and apoptosis [21]. Furthermore, Nishida et al. have shown the involvement of PI3K/AKT in the regulation of platelet-derived growth factor-induced PSC migration [10,18].

Moreover, most of the biological processes, including cell proliferation, survival, migration, or protein secretion, are driven by intracellular Ca^2+^, which acts as a universal second messenger. A few studies have revealed that intracellular Ca^2+^ signaling is also crucial for the regulation of PSC physiology. Won et al. were the first to demonstrate that Ca^2+^ signaling is different between quiescent and activated PSCs. Activated PSCs were characterized by transient elevations of intracellular Ca^2+^ in response to thrombin or trypsin, which were absent in quiescent PSCs. They have also reported that nuclear Ca^2+^ signals are essential for promoting activated PSC proliferation [22]. Therefore, one of the major Ca^2+^ entry pathways in non-excitable cells is the store-operated calcium channels (SOCs), also known as Ca^2+^ release-activated Ca^2+^ (CRAC) channels. SOCs are mainly composed of the pore-forming Orai1 protein and the Ca^2+^ -sensing stromal interaction molecule STIM1, and they become activated after endoplasmic reticulum (ER) Ca^2+^ depletion [23]. A recent study has pharmacologically identified the presence of CRAC channels in mice PSCs [24]. These data were supported by Waldron et al., who demonstrated the expression of Orai1, Orai2, and STIM1 in mice PSCs [25].

Although many studies have been focused on the molecular and cellular mechanisms of PSC activation, a very limited number of data is available on the role of Ca^2+^ and SOCs channels in the regulation of PSC activation and the associated signaling pathways. In the present study, we focused on two principal PSC activation hallmarks, (i) cell proliferation and (ii) cytokine secretion, particularly TGF_β1_ secretion. We aimed to investigate the role of Ca^2+^ entry through the Orai1 channel in the regulation of these two processes and the associated molecular mechanisms. We showed, for the first time, that the Orai1 channel is expressed and functional in human-activated PSCs. Moreover, we demonstrated that Orai1-Ca^2+^ entry is essential for human-activated PSC proliferation and TGF_β1_ secretion by triggering the activation of the AKT signaling pathway. Interestingly, TGF_β1_ treatment induced an autocrine positive feedback loop, which led to the enhancement of PSC Orai1/AKT-dependent proliferation through the regulation of Orai1 activity and expression.

## 2. Results

### 2.1. Functional Expression of Orai1 Channel in PS-1 and RLT Human-Activated PSCs

**Figure 1:**
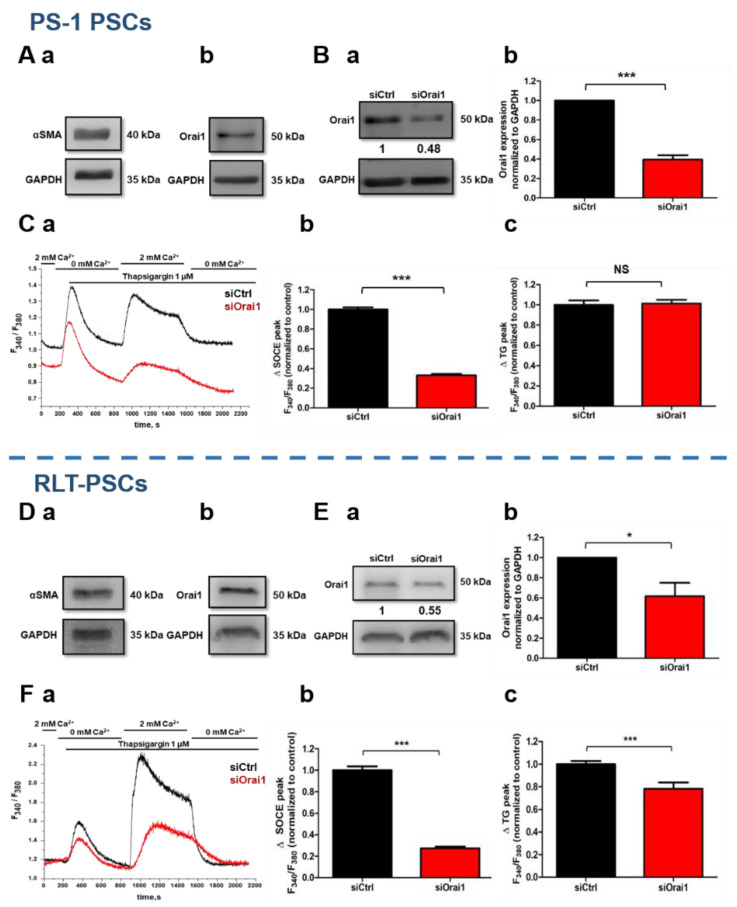
Orai1 channel is functionally expressed in human-activated PSCs by regulating the store-operated Ca^2+^ entry (SOCE). (**A**,**D**) Protein expression of αSMA, the principal marker of PSC activation (**Aa**,**Da**), and Orai1 channel (**Ab**,**Db**), by Western blot, 72 h post-proliferation, in PS-1 (**A**) and RLT (**D**) human-activated PSCs. (**B**,**E**) Evaluation of Orai1 siRNA 72 h post-transfection efficiency by Western blot. Illustrative Western blot of Orai1 protein inhibition (**Ba**,**Ea**), with the quantification (**Bb**, **Eb**), in both PS-1 (**B**) and RTL (**E**) PSCs. Western blotting results were first normalized to the referent protein GAPDH and then to the control (N = 3, * *p* < 0.05, *** *p* < 0.001, Student’s *t-*test). (**C**,**F**) Assessment of Orai1 channel’s function in siOrai1 transfected PS-1 (**C**) and RLT (**F**) cells, using calcium imaging, 72 h post-transfection. SOCE was measured after endoplasmic reticulum release induced by 1 µM of thapsigargin, illustrated by representative traces (**Ca**,**Fa**) of SOCE measurements in both cell lines. All histograms are represented as the average ± SEM normalized to the control, of SOCE (siCtrl: *n* = 210, siOrai1: *n* = 189, *N* = 5 for PS-1 cells, (**Cb**)) (siCtrl: *n* = 91, siOrai1: *n* = 80, *N* = 3 for RLT cells, (**Fb**)), and of endoplasmic reticulum release induced by 1 µM Thapsigargin (siCtrl: *n* = 210, siOrai1: *n* = 184, *N* = 5 for PS-1 cells, (**Cc**)) (siCtrl: *n* = 91, siOrai1: *n* = 80, N = 3 for RLT cells, (**Fc**)) (* *p* < 0.001, NS: no significant, Student’s *t-*test, n: number of cells, N: number of passage).

At first, the activated state of PS-1 and RLT human immortalized PSCs was determined by the presence of αSMA expression, the main activation marker of PSCs, using Western blot experiments (Figure 1(Aa,Da), respectively). We, therefore, investigated Orai1 channel expression, also by Western blotting, which revealed its presence in the PS-1 (Figure 1(Ab)) and RLT (Figure 1(Db)) human-activated PSCs, and by immunofluorescence staining for PS-1 (Figure S1A).

To establish whether Orai1 channels are functional too in PS-1 and RLT cells, we performed Ca^2+^ - imaging experiments using Fura2/AM fluorescent probe. Since, it is well known that Orai1 is one of the key players of store-operated Ca^2+^ entry (SOCE) in the majority of cell types, we evaluated this possibility by using the siRNA approach. Orai1 protein expression was decreased by 60.54 ± 4.40% in PS-1 cells (*N* = 3, *p* < 0.001, Figure 1(Ba,Bb)), and by 38.38 ± 13.26% in RLT cells (*N* = 3, *p* < 0.05, Figure 1(Ea,Eb)), 72 h post-transfection. We then measured the SOCE after an endoplasmic reticulum (ER) Ca^2+^ depletion induced by thapsigargin (Tg), which acts as an irreversible inhibitor of the sarco/endoplasmic reticulum Ca^2+^-ATPase (SERCA) pump. Orai1 silencing reduced SOCE by 67.81% (siCtrl: 100 ± 2.52%, siOrai1: 32.19 ± 1.36%, *N* = 5, *p* < 0.001, Figure 1(Ca,Cb)) in PS-1 cells, and by 72.65% (siCtrl: 100 ± 3.54%, siOrai1: 27.35 ± 1.55%, *N* = 3, *p* < 0.001, Figure 1(Fa,Fb)) in RLT cells. Moreover, in both, PS-1 and RLT cells, Orai1 knocking down decreased the Ca^2+^ basal fluorescence ratio, by 5.93% (siCtrl: 100 ± 1.25%, siOrai1: 94.07 ± 0.84%, *N* = 5, *p* < 0.001, Figure S1(Ba)) and by 17.17% (siCtrl: 100 ± 1.42% siOrai1: 82.83 ± 1.51%, *N* = 3, *p* < 0.001, Figure S1(Bb)), respectively. However, contrary to PS-1 cells (siCtrl: 100 ± 4.21%, siOrai1: 101.35 ± 3.53%, *N* = 5, Figure 1(Ca,Cc)), Orai1 inhibition led to 21.80% reduction in the Ca^2+^ ER depletion induced by Tg, in RLT cells (siCtrl: 100 ± 2.61%, siOrai1: 78.20 ± 5.60%, *p* < 0.001, *N* = 3, Figure 1(Fa,Fc)).

These results showed that the Orai1 channel is functionally expressed, and it participates in the SOCE as well as the regulation of Ca^2+^ basal concentration in human-activated PSCs.

To investigate whether SOCE is already induced by the PS-1 cell culture conditions (with cell medium containing 10% fetal bovine serum (FBS)), we performed Ca^2+^ imaging experiments after overnight FBS deprivation. Indeed, it is well known that FBS can induce a Ca^2+^ ER depletion by acting on the inositol triphosphate receptor, which will promote STIM1 translocation to the cell membrane in order to activate Orai1 protein and so permit the SOCE [26,27,28,29]. We so measured SOCE after perfusion of 1 µM Tg and 10% FBS, and we observed that both Tg and FBS-induced Ca^2+^ ER depletion triggering a SOCE. However, FBS stimulation led to a lower SOCE (30% decrease) compared to Tg stimulation (Tg: 100 ± 2.94%, FBS: 73.41 ± 2.37%, *N* = 3, *p* < 0.001, Figure S1(Ca,Cb)). These data revealed that, in our culture conditions, Orai1 is activated and leads to SOCE that regulates Ca^2+^ basal concentration.

### 2.2. Orai1 Is Involved in Human-Activated PSC Proliferation and TGF_β1_ Secretion

**Figure 2:**
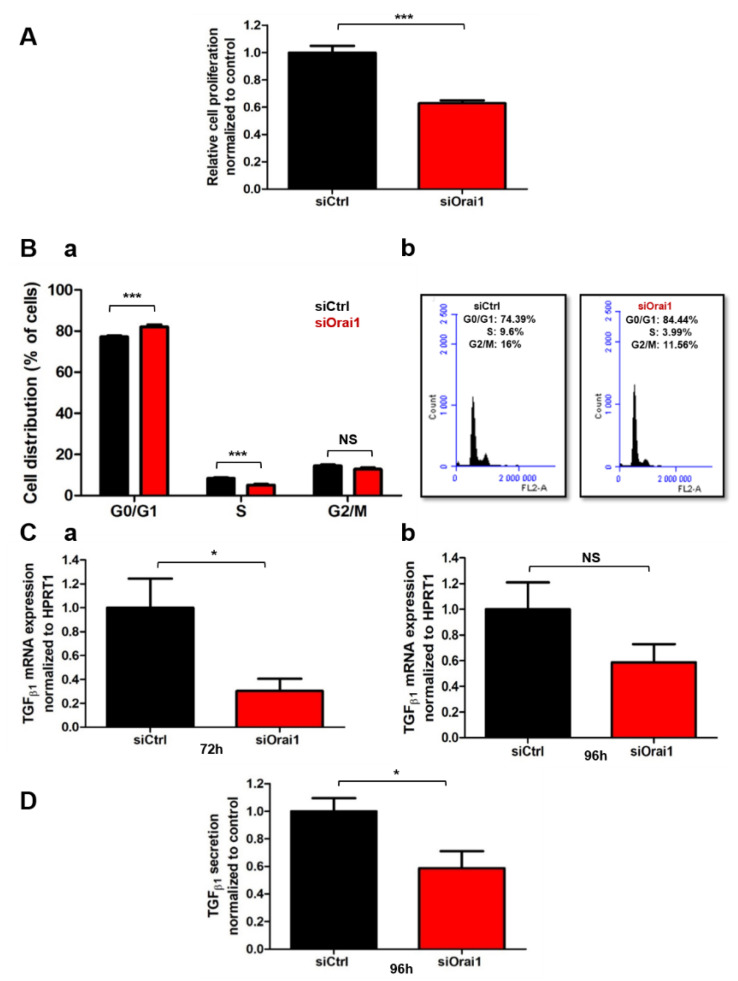
Orai1 modulates human-activated PSC proliferation and TGF_β__1_ secretion. (**A**) Effect of Orai1 silencing on PS-1 cell proliferation, assessed 72 h post-transfection by MTT assay (*** *p* < 0.001, *N* = 4, Student’s *t-*test). (**B**) Implication of Orai1 in PS-1 cell cycle progression. Cell distribution (G0/G1, S and G2/M phase) was examined by flow cytometry 72 h after transfection, with propidium iodide staining (*** *p* < 0.001, NS: no significant, *N* = 3, two-way ANOVA followed by Bonferroni *post hoc* test, (**Ba**) and represented by an illustrative cell cycle profile 72 h after Orai1 inhibition (**Bb**). (**C**) Impact of Orai1 knocking down on TGF_β__1_ mRNA expression, evaluated by qPCR, 72 h (**Ca**) and 96 h (**Cb**) post-transfection. (**D**) Role of Orai1 inhibition on TGF_β__1_ secretion, assessed by ELISA assay, 96 h post-transfection (* *p* < 0.05, *N* = 3, Student *t-*test). Values were normalized to control and reported as mean ± SEM.

We next wondered whether the Orai1 channel could drive some of PSCs activation processes. We chose to focus on PSC proliferation and cytokine secretion, which are two of the main activation hallmarks of PSCs. After 72h of Orai1 silencing, we assessed PS-1 and RLT’s cell proliferation by MTT colorimetric assay and we found that Orai1 knock-down decreased by 37.12% PS-1’s cell proliferation rate (siCtrl: 100 ± 4.89%, siOrai1: 62.88 ± 2.04%, *N*= 4, *p* < 0.001, Figure 2A), and by 31.39% RLT’s cell proliferation (siCtrl: 100 ± 3.80%, siOrai1: 68.61 ± 6.21%, *N* = 3, *p* < 0.001, Figure 2A). We next performed cell cycle analysis on PS-1 cells using flow cytometry (Figure 2(Ba,Bb)). Orai1 silencing induced a cell cycle arrest in G0/G1 phase (siCtrl: 77.18 ± 0.65%, siOrai1: 82.10 ± 1.06%, *N* = 3, *p* < 0.001), followed by a decrease in cell number in S phase (siCtrl: 8.37 ± 0.37%, siOrai1: 5.12 ± 0.46%, *N* = 3, *p* < 0.01), without affecting the G2/M phase (siCtrl: 14.44 ± 0.61%, siOrai: 12.76 ± 0.80%, *N* = 3, Figure 2(Ba,Bb)). Simultaneously, we checked if the observed effect may be caused by an increase of cell mortality, which was evaluated by Trypan blue colorimetric assay. In both PS-1 and RLT PSCs, we did not reveal any significant effect on cell mortality (Figure S2C,D). In accordance with this data, apoptosis analysis on PS-1 cells, using the Annexin V/IP staining, confirmed the absence of any effect on cell mortality (Figure S2(Ea,Eb)).

Another characteristic of PSC activation is the abundant secretion of TGF_β1_ multifunctional cytokine [6,30]. To test whether Orai1 could be involved in the modulation of TGF_β1_ expression and secretion, we first quantified TGF_β1_ mRNA expression after 72 h of Orai1 protein inhibition in both PS-1 and RLT cells. Orai1 knock-down led to 69.71% TGF_β1_ mRNA decrease in PS-1 cells (siCtrl: 100 ± 24.45%, siOrai1: 30.29 ± 10.25%, *N* = 3, *p* < 0.05, Figure 2(Ca)), and to 53.95% mRNA decrease in RLT cells (siCtrl: 100 ± 16.87%, siOrai1: 46.05 ± 12.59, *N* = 3, *p* < 0.05, Figure S2B). We then evaluated the impact of Orai1 in the TGF_β1_ secretion process by ELISA assay (Figure 2D) in PS-1 cells. We observed no effect on TGF_β1_ secretion 72 h after Orai1 silencing, but we found a 41.31% decrease in TGF_β1_ secretion after 96 h of siOrai1 transfection (siCtrl: 100 ± 9.49%, siOrai1: 58.69 ± 12.30%, *N* = 3, *p* < 0.05, Figure 2D). Simultaneously, we have also looked at TGF_β1_ mRNA expression to see whether the effect of Orai1 silencing persists 96 h post-transfection. However, even though we observed a tendency of TGF_β1_ mRNA decrease, it remained no significant (siCtrl: 100 ± 20.95%, siOrai1: 58.67 ± 14.19%, *N* = 3, Figure 2(Cb)).

These results demonstrated the involvement of the Orai1 channel in human-activated PSC proliferation by regulating cell cycle progression in the G1 phase and G1/S transition and TGF_β1_ secretion.

### 2.3. Orai1 Regulates the Activation of AKT But Not of ERK1/2 and SMAD2 in Human-Activated PSCs

**Figure 3:**
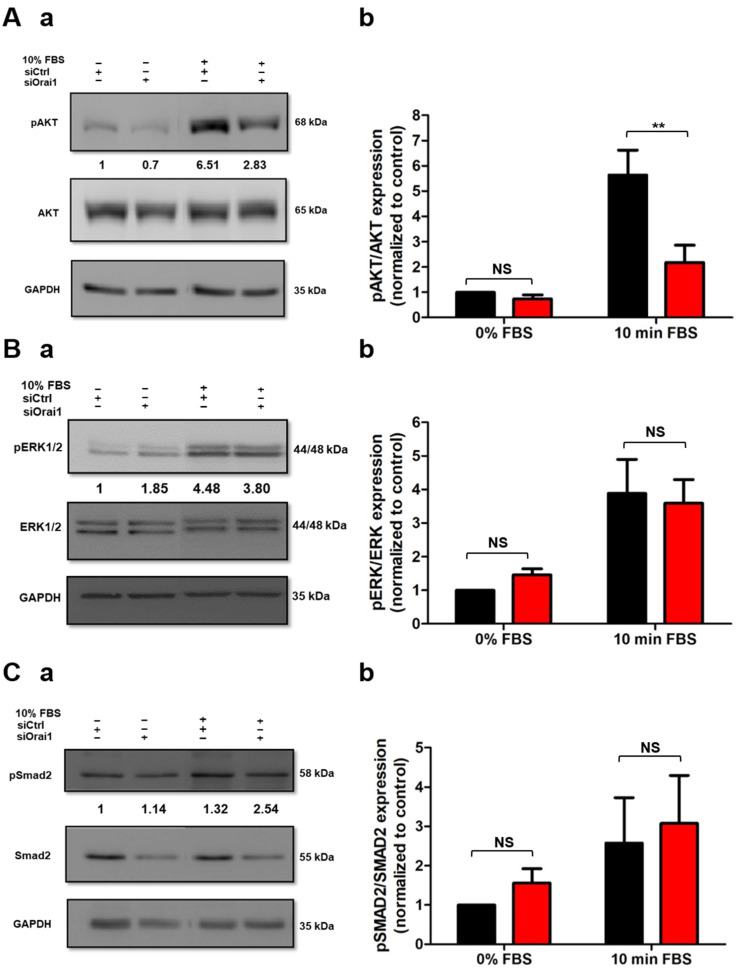
Orai1 mediates AKT activation but not ERK1/2 nor SMAD2 phosphorylation in human-activated PSCs. (**A**) Involvement of Orai1 in AKT phosphorylation in PS-1 cells. Representative Western blot showing the effect of Orai1 inhibition after FBS starvation of transfected cells overnight. Cells were then stimulated 10 min with FBS to evaluate the impact of Orai1 on AKT activation (**Aa**). AKT phosphorylation was quantified by the ratio of phosphorylated AKT form/total AKT protein (siCtrl + 10 min FBS: 5.63 ± 0.99-fold, siOrai1 + 10 min FBS: 2.18 ± 0.69-fold, (**Ab**). (**B**) Assessment of ERK1/2 activation after Orai1 knocking down in PS-1 cells. Representative Western blot showing the effect of Orai1 silencing on ERK1/2 activation, using the protocol described above (**Ba**). ERK1/2 phosphorylation was quantified by the ratio of phosphorylated ERK1/2 form/total ERK1/2 protein (**Bb**). (**C**) Evaluation of Orai1 silencing on SMAD2 phosphorylation. Representative Western blot showing the effect of siOrai1 transfected cells on SMAD2 activation (**Ca**), with the quantification using the ratio of phosphorylated SMAD2 form/total SMAD2 protein (**Cb**). All values were first normalized to the referent protein GAPDH and then to the 0% FBS control condition. All experiments were realized 72 h post-transfection. Values were reported as mean ± SEM (** *p* < 0.01, NS, at least N = 3, two-way ANOVA followed by Bonferroni *post hoc* test).

Since the discovery of PSCs, accumulating studies have been focused on the investigation of the signal transduction pathways implicated in PSC activation [10,18]. AKT, ERK1/2, and SMAD2 phosphorylation have been reported to be three of the main signaling pathways involved in PSC activation processes [18,31,32,33]. Hence, we sought to determine whether Orai1 regulates one of these signaling pathways. We, therefore, evaluated AKT, ERK1/2, and SMAD2 phosphorylation levels after 72 h of siOrai1 transfection, by Western blotting, in both PS-1 (Figure 3) and RLT cells (Figure S4). Silencing of Orai1 induced a 61.4% decrease in AKT phosphorylation after 10% FBS stimulation (Figure 3(Aa,Ab)), without affecting the AKT total protein amount (Figure S3A) in PS-1 cells. Similar experiments performed on ERK1/2 and SMAD2 activation showed that Orai1 silencing failed to affect ERK1/2 (Figure 3(Ba,Bb)) and SMAD2 (Figure 3(Ca,Cb)) phosphorylation as well as ERK1/2 total protein amount (Figure S3B). However, under 0% FBS conditions, the SMAD2 total protein amount was decreased in the siOrai1 transfected cells, without any alteration in 10% FBS conditions (Figure S3C). Interestingly, 72 h Orai1 knocking down led to a 46.8% reduction of AKT phosphorylation, in RLT cells, without modifying ERK1/2 and SMAD2 activation, and either the total protein amount of each one (Figure S4A–C).

### 2.4. AKT Signaling Pathway Mediates Human-Activated PSC Proliferation and TGF_β1_ Secretion

**Figure 4:**
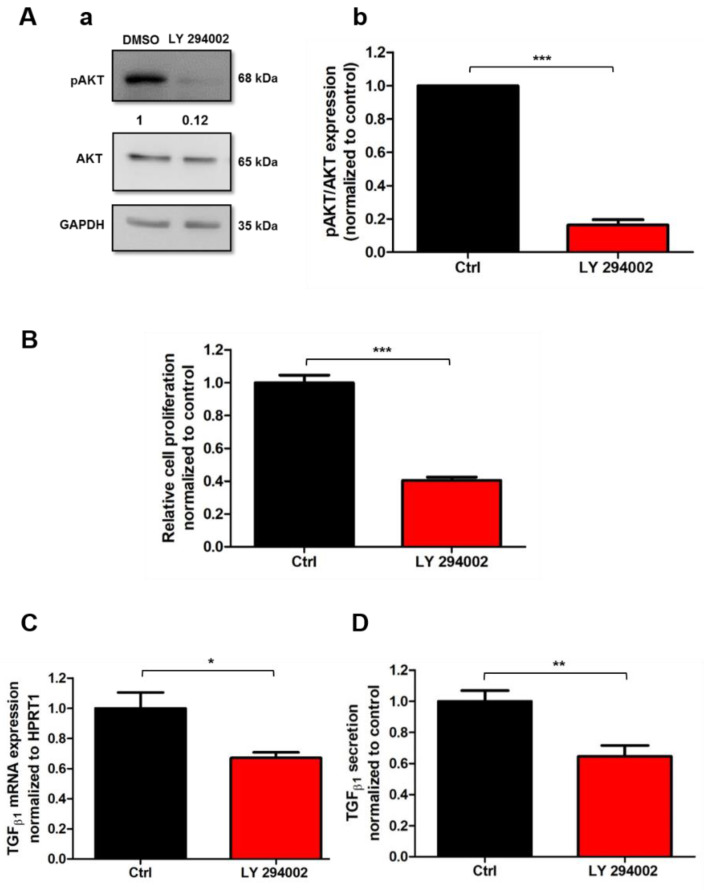
AKT signaling pathway controls PSC proliferation and TGF_β__1_ secretion. (**A**,**B**) Inhibition of AKT activation after 72 h cell treatment with the PI3K/AKT pharmacological inhibitor LY 294002 (20 µM) decreased PS-1 cell proliferation. Representative Western blot of AKT reduced phosphorylation by LY 294002 treatment (**Aa**) and the quantification as previously described (**Ab**) (N = 3, *** *p* < 0.001, Student *t-*test). All values were first normalized to the referent protein GAPDH and then to the control condition, reported as mean ± SEM. Cell proliferation was evaluated after 72 h of treatment by MTT assay (N = 4, *** *p* < 0.001, Student *t-*test) (**B**). (**C**,**D**) Effect of AKT inhibition on TGF_β__1_ mRNA expression (**C**) and secretion (**D**) after 72 h LY 294002 treatment (* *p* < 0.05, ** *p* < 0.01, N = 3, Student *t-*test). Values were normalized to control and reported as mean ± SEM.

Up until now, very few studies have demonstrated the involvement of the PI3K/AKT signaling pathway in PSC proliferation, without showing a direct role in the regulation of this process [21]. We thus investigated the potential direct implication of the AKT pathway in the modulation of PS-1 proliferation. To determine this, we treated PS-1 for 72 h with the pharmacological inhibitor of PI3K/AKT pathway LY 294002 and evaluated the effect on both AKT phosphorylation and cell proliferation. LY 294002 treatment drastically reduced AKT activation by 83.68 ± 3.20% (*N* = 3, *p* < 0.001, Figure 4(Aa,Ab)). PS-1 treatment with LY 294002 induced a 59.58% decrease in the proliferation rate, revealing that AKT pathway mediates PS-1 cell proliferation (Ctrl: 100 ± 4.60%, LY 294002: 40.42 ± 2.06%, *N* = 4, *p* < 0.001, Figure 4B).

Moreover, some studies established the involvement of the AKT pathway in TGF_β1_ mRNA and protein expression in hepatic stellate cells, suggesting that TGF_β1_ might be regulated by the AKT pathway [34]. However, there is no evidence yet of this mechanism in PSCs. We then assessed whether the AKT pathway plays a role in TGF_β1_ mRNA expression and secretion using the pharmacological inhibitor LY 294002. We observed a 32.96% reduction in TGF_β1_ mRNA (Ctrl: 100 ± 10.44%, LY 294002: 67.04 ± 3.75%, N = 3, *p* < 0.05) after the pharmacological inhibition of AKT pathway (Figure 4C). These results were completed with the evaluation of TGF_β1_ secretion using the ELISA assay. In accordance with the mRNA transcripts, LY 294002 treatment induced 35.41% decrease in TGF_β1_ secretion (Ctrl: 100 ± 6.83%, LY 294002: 64.59 ± 6.92%, N = 3, *p* < 0.01, Figure 4D)

Taken together, these data suggested that Orai1 regulates PSC proliferation and TGF_β1_ secretion, probably through the activation of the AKT signaling pathway.

### 2.5. TGF_β__1_ Promotes Orai1-Mediated Ca^2+^ Entry and Increases Both Orai1 mRNA and Protein Expression in Human-Activated PSCs

**Figure 5:**
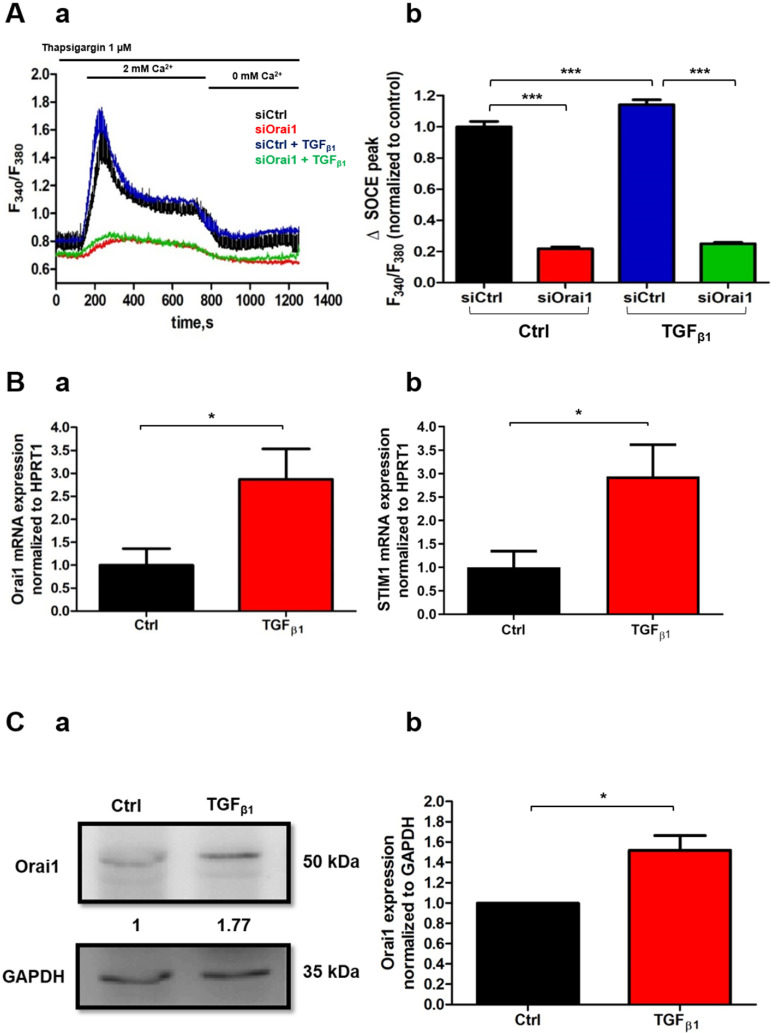
TGF_β__1_ enhances Orai1-mediated Ca^2+^ entry and increases both Orai1 mRNA and protein expression. (**A**) TGF_β__1_ (5 ng/mL) perfusion was applied to investigate the effect of TGF_β__1_ on Orai1 activity. siCtrl and siOrai1 72 h-transfected cells were pre-treated for 5 min with 1 µM of Thapsigargin before use for SOCE measurements in Ca^2+^ - imaging. Illustrative traces of SOCE measurements (**Aa**) and the quantification of perfused and non-perfused siCtrl and siOrai1 transfected cells (siCtrl: n = 133, siCtrl + TGF_β__1_: n = 163, siOrai1: n = 102, siOrai1 + TGF_β__1_: n = 122, N = 3, *** *p* < 0.001, one-way ANOVA followed by Bonferroni multiple comparison test, n: number of cells, N: number of passages, (**Ab**). All values were normalized to siCtrl non-treated with TGF_β1_ condition and reported as mean ± SEM. (**B**,**C**) Impact of 48 h TGF_β__1_ treatment (20 ng/mL) in the presence of low-FBS conditions (1%) on Orai1 and STIM1 (**B**) mRNA and Orai1protein expression (**C**). Quantification of Orai1 (**Ba**) and STIM1 (**Bb**) mRNA transcripts 48 h post-TGF_β__1_ treatment (* *p* < 0.05, N = 3, Student *t*-test) (**B**). Representative Western blot showing the effect of TGF_β__1_ treatment on Orai1 expression (**Ca**) with the quantification (**Cb**) (* *p* < 0.05, N = 3, Student *t*-test). All values were first normalized to the referent protein GAPDH and then to the control condition, reported as mean ± SEM.

TGF_β1_ has been reported to be involved in the fibrosis mediated by PSC activation [35] and the autocrine regulation of PSCs [15]. Several reports have already demonstrated the involvement of TGF_β1_ in the regulation of some cell types’ intracellular mechanisms, including lung, embryonic fibroblasts, and pancreatic cancer cells, through a Ca^2+^ -dependent pathway [36,37,38]. Indeed, it has been shown for a long time that TGF_β1_ can modulate Ca^2+^ signaling by stimulating the Ca^2+^ influx and thus by increasing the cytoplasmic Ca^2+^ concentration [36]. According to these findings, we wondered whether TGF_β1_ could also mediate the Ca^2+^ influx in PS-1 human-activated PSCs by promoting Orai1-mediated Ca^2+^ entry. To evaluate this hypothesis, we performed Ca^2+^ imaging experiments where we perfused TGF_β1_ on siCtrl and siOrai1 transfected cells and measure the SOCE response. Interestingly, we noted a 14% increase in SOCE after TGF_β1_ perfusion on siCtrl transfected cells compared to the siCtrl non-perfused with TGF_β1_ cells. However, we did not remark any difference in Orai1 knock-down cells, perfused and non-perfused with TGF_β1_, inferring an effect of TGF_β1_ on Orai1-mediated Ca^2+^ entry (siCtrl: 100 ± 3.33%, siCtrl+ TGF_β1_: 114.14 ± 3.07%, siOrai1: 21.74 ± 1.19%, siOrai1+ TGF_β1_: 24.96 ± 0.92%, N = 3, *p* < 0.001, *p* > 0.05, Figure 5(Aa,Ab)). We then supposed that the short-term effect of TGF_β1_ on Orai1-mediated Ca^2+^ entry might be due to a long-term effect of this cytokine on Orai1 mRNA and protein expression. We thus treated PS-1 cells for 48 h with TGF_β1_, in the presence of low-FBS conditions (1%), and we observed that both, Orai1 mRNA and protein expression were increased following TGF_β1_ treatment. qPCR experiments revealed a 2.87-fold increase in Orai1 mRNA transcripts in TGF_β1_ treated cells (Ctrl: 1 ± 0.36-fold, TGF_β1_: 2.87± 0.66-fold, N = 3, *p* < 0.05, Figure 5(Ba)). Furthermore, it is known that the store-operated Orai1-mediated Ca^2+^ entry is promoted by the intracellular STIM1 protein. Consequently, we quantified in parallel the impact of TGF_β1_ treatment on STIM1 mRNA expression, finding a comparable increase in STIM1 mRNAs as for Orai1 (Ctrl: 1 ± 0.34-fold, TGF_β1_: 2.94 ± 0.68-fold, N = 3, *p* < 0.05, Figure 5(Bb)). These data suggest that TGF_β1_ stimulates store-operated Orai1-mediated Ca_2+_ entry modulating the expression of both main SOCE actors, Orai1 and STIM1. Similarly, Western blotting experiments showed a 1.52 ± 0.15-fold rise of Orai1 protein level in treated compared to non-treated with TGF_β1_ cells (N = 3, *p* < 0.05 Figure 5(Ca,Cb)).

### 2.6. TGF_β1_ Enhances Orai1/AKT-Dependent Proliferation Through an Autocrine Positive Feedback Loop in Human-Activated PSCs

**Figure 6:**
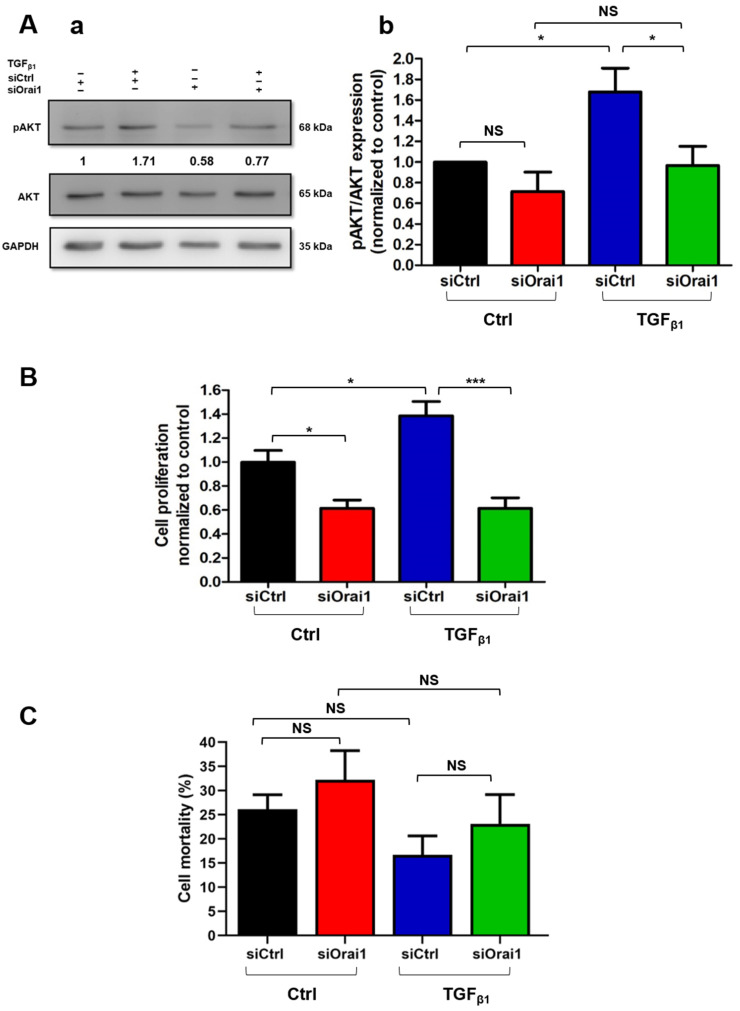
TGF_β1_ promotes Orai1/AKT-dependent proliferation through an autocrine positive feedback loop in human-activated PSCs. (**A**) TGF_β1_ stimulation decreased AKT phosphorylation in Orai1 knocked-down cells. Orai1 knocked-down cells were FBS-starved overnight and then stimulated 30 min with TGF_β1,_ 72 h post-transfection. Representative Western blot of TGF_β1_ stimulation on Orai1 knocked-down mediated AKT activation (**Aa**) and the quantification (* *p* < 0.05, at least N = 3, two-way ANOVA followed by Bonferroni *post hoc* test, (**Ab**). All values were first normalized to the referent protein GAPDH and then to siCtrl non-treated with TGF_β1_ condition and reported as mean ± SEM. (**B**,**C**) Involvement of TGF_β1_ in PS-1 cell proliferation and survival. Transfected cells were treated 48 h with TGF_β1_ (20 ng/mL) within an FBS-free medium, and the proliferation rate, as well as the mortality rate, were evaluated by Trypan blue assay, 72 h post-transfection (* *p* < 0.05, *** *p* < 0.001, NS, N = 3, one-way ANOVA followed by Bonferroni multiple comparison tests). All values were normalized to siCtrl non-treated with TGF_β1_ condition and reported as mean ± SEM. Cell mortality was calculated using the formula: % of cell death = number of dead cells/number of total cells, reported as mean ± SEM for each condition.

We thereafter wondered whether TGF_β1_ could also be involved in the promotion of the Orai1/AKT-dependent proliferation. Indeed, TGF_β1_ is known to have a dual role in cell proliferation, since according to the cell type, it can induce an inhibition or stimulation of cell proliferation. Furthermore, it has been shown to enhance the hepatic stellate cell’s proliferation, known as counterparts of PSCs [34]. However, its role in PSC proliferation and proliferation-related signaling pathways remains little know. We hence started by assessing the impact of TGF_β1_ on Orai1-mediated AKT activation after inhibiting Orai1 expression. For that, we FBS-starved siCtrl and siOrai1 cells overnight and then stimulated them 30 min with TGF_β1_ (Figure 6(Aa,Ab)). As expected, TGF_β1_ stimulation increased by 1.68 ± 0.23-fold (N = 4, *p* < 0.05, Figure 6(Ab)) the AKT phosphorylation in the siCtrl cells compared to the no-stimulated siCtrl cells. Moreover, we revealed a 42.37% decrease in AKT activation in siOrai1 (0.97 ± 0.18-fold) compared to siCtrl stimulated with TGF_β1_ cells (N = 4, *p* < 0.05, Figure 6(Ab)), whereas no significant effect was observed in siOrai1 stimulated and no-stimulated with TGF_β1_ cells. These results inferred the involvement of TGF_β1_ in the stimulation of Orai1-mediated AKT phosphorylation.

Therefore, we wanted to evaluate if TGF_β1_ promotion of Orai1-mediated AKT activation regulates PS-1 cell proliferation or survival. After treating siCtrl and siOrai1 transfected cells 48 h with TGF_β1_, we found that TGF_β1_ enhanced by 38.55% the cell proliferation of siCtrl cells compared to the non-treated ones (N = 3, *p* < 0.05, Figure 6B). In addition, TGF_β1_ treatment did not reveal any effect on the proliferation rate of siOrai1 cells compared to the non-treated siOrai1 cells (siCtrl: 100 ± 9.63%, siCtrl + TGF_β1_: 138.55 ± 11.91%, siOrai1: 61.45 ± 6.85%, siOrai1 + TGF_β1_: 61.45 ± 8.81, N = 3, *p* > 0.05, Figure 6B). Simultaneously, the Trypan blue colorimetric assay showed the no involvement of TGF_β1_ in PS-1’s cell mortality and so in cell survival (siCtrl: 26.18 ± 2.97%, siCtrl + TGF_β1_: 16.70 ± 3.92%, siOrai1: 32.22 ± 6.05%, siOrai1 + TGF_β1_: 23.11 ± 6.07, N = 3, *p* > 0.05, Figure 6C). It should be noted that the impact of TGF_β1_ on these two cellular processes was investigated under 0% FBS conditions since the 1% FBS was still preventing its action on cell proliferation and survival Hence, 0% FBS conditions induced an elevation of the basal cell death.

TGF_β1_ is also known to be an important profibrotic factor due to its ability to regulate αSMA expression, the main activation marker of PSCs [15]. Furthermore, it has been shown that ion channels, particularly TRPC6, can regulate αSMA expression as well, in human-activated hepatic stellate cells, counterparts of PSCs [39], and human intestinal myofibroblasts [40]. In these later, TRPC6 interacts with αSMA, too, to form a protein complex. According to these data, we hypothesized that Orai1 could modulate αSMA expression or form a complex with it. However, Orai1 silencing failed to induce any significant decrease in αSMA protein expression (*p* > 0.05, N = 3, Figure S5(Aa,Ab)), and neither to interact with αSMA (N = 3, Figure S5(Ba,Bb)). To confirm these findings, we next investigated whether Orai1-mediated TGF_β1_ expression and secretion might be involved in the regulation of αSMA expression. We thereby treated siCtrl and siOrai1 transfected cells 48h with TGF_β1_, in the presence of low-FBS conditions. As expected, TGF_β1_ treatment of siCtrl cells induced a 3.82 ± 0.54-fold increase in αSMA expression, as quantified by Western blotting, compared to siCtrl non-treated cells (*p* < 0.05, N = 4, Figure S5(Ca,Cb)). However, treatment of siOrai1 transfected cells with TGF_β1_ did not reveal any significant difference in αSMA expression compared to the siCtrl TGF_β1_ treated cells (*p* > 0.05, N = 4, Figure S5(Ca,Cb)), indicating that Orai1-induced TGF_β1_ expression and secretion is not implicated in the modulation of αSMA expression.

Based on these data, we can conclude that TGF_β1_ is involved in an autocrine positive feedback loop by the promotion of Orai1-dependent AKT phosphorylation, leading to PS-1 cell proliferation enhancement through the stimulation of Orai1-mediated Ca^2+^ entry and the increase in Orai1 mRNA and protein expression.

### 2.7. Ca^2+^ Entry Through Orai1 Regulates AKT-Dependent Proliferation and TGF_β1_ Secretion in PS-1 Human-Activated PSCs

**Figure 7:**
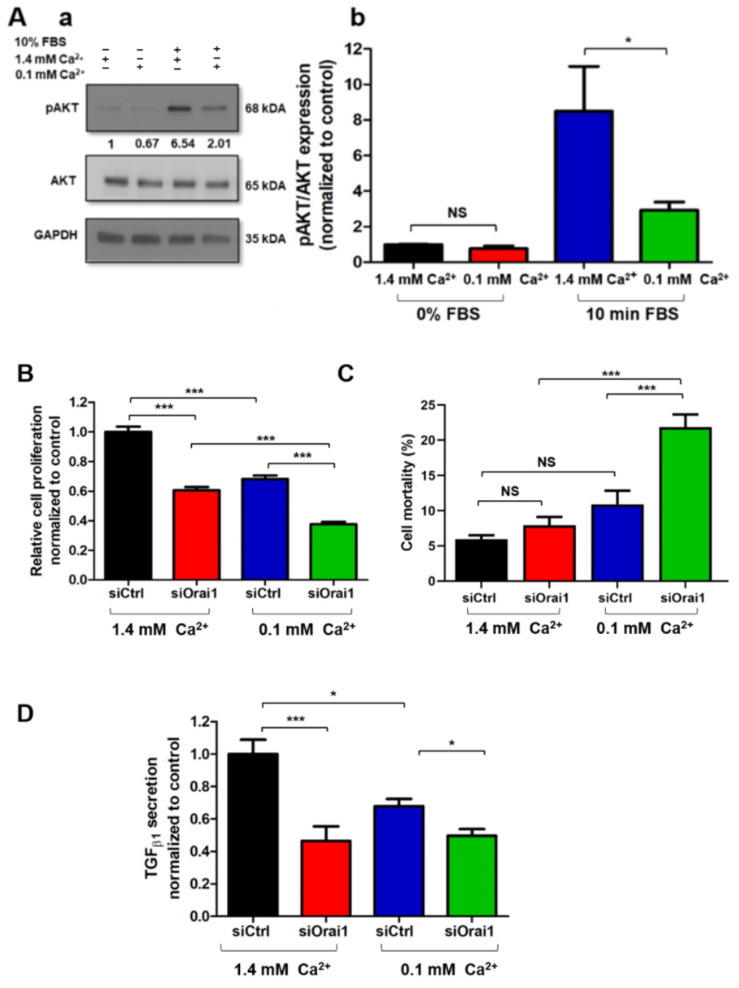
Ca^2+^ entry through Orai1 regulates AKT-dependent proliferation and TGF_β__1_ secretion in PS-1 human-activated PSCs. (**A**) Effect of extracellular Ca^2+^ decrease on AKT activation, 72 h post-proliferation, after overnight FBS starvation of no transfected cells. Then PS-1 cells were pre-treated for 30 min with 1.3 mM EGTA in order to reduce the extracellular Ca^2+^ concentration (0.1 mM Ca^2+^ ), with (+) and without (-) 10 min of 10% FBS stimulation. Representative Western blot of low extracellular Ca^2+^ concentration impact on AKT phosphorylation (**Aa**). AKT activation was evaluated as described previously, by the ratio of phosphorylated AKT form/total AKT protein (1.4 mM Ca^2+^ +10 min FBS: 8.5 ± 2.52-fold, 0.1 mM Ca^2+^ + 10 min FBS: 2.92 ± 0.46-fold, N = 3, * *p* < 0.05, NS, two-way ANOVA followed by Bonferroni *post hoc* test, (**Ab**). Values were first normalized to the referent protein GAPDH and then to the 0% FBS + 1.4 mM Ca^2+^ condition, reported as mean ± SEM. (**B**) Evaluation of siOrai1 transfected PS-1 cells’ proliferation after 72 h of 1.3 mM EGTA treatment by MTT assay. (**C**) Impact of Orai1 knocked-down cells, treated with low Ca^2+^ condition, on cell survival, assessed by Trypan blue assay. Cell mortality was calculated using the formula: % of cell death = number of dead cells/number of total cells, reported as mean ± SEM for each condition. (**D**) Similarly, TGF_β__1_ secretion was measured in Orai1 knocked-down PS-1 cells, in the presence of low-Ca^2+^ conditions, by ELISA assay. All values were normalized to 1.4 mM Ca^2+^ control condition (except for the mortality rate) and reported as mean ± SEM (*** *p* < 0.001, * *p* < 0.05, NS, N = 3, one-way ANOVA followed by Bonferroni multiple comparison test).

It is well known that most of the signaling pathways are regulated by the Ca^2+^ second messenger. Moreover, it has been demonstrated that Ca^2+^ modulates a myriad of biological processes, including cell proliferation or cytokine secretion [41,42]. To determine whether Ca^2+^ and, more specifically, Ca^2+^ entry through Orai1 regulates AKT activation, as well as Orai1/AKT-dependent proliferation and TGF_β1_ secretion in PS-1 cells, we reduced the physiological extracellular Ca^2+^ concentration. We started by evaluating the impact of extracellular Ca^2+^ modification on AKT phosphorylation after overnight FBS starvation of PS-1 non-treated cells. As expected, 30 min treatment with low Ca^2+^ -conditions (0.1 mM Ca^2+^ ) led to 65.60% decrease in AKT activation, compared to the physiological Ca^2+^ -conditions (1.4 mM Ca^2+^ ) (N = 3, *p* < 0.05, Figure 7(Aa,Ab)).

We thereafter wanted to establish whether Orai1-mediated Ca^2+^ entry is involved in the modulation of PS-1 cell proliferation and TGF_β1_ secretion. We thus incubated siCtrl and siOrai1 transfected cells for 72 h with low and physiological Ca^2+^ -conditions. Interestingly, we noticed a more important effect on the proliferation rate of siOrai1 cells treated with low Ca^2+^ -conditions compared to the ones in physiological Ca^2+^ -conditions (siCtrl 1.4 mM Ca^2+^: 100 ± 3.45%, siCtrl 0.1 mM Ca^2+^: 68.29 ± 2.27%, siOra1 1.4 mM Ca^2+^: 60.58 ± 2.19%, siOrai1 0.1 mM Ca^2+^: 37.69 ± 1.59, N = 3, *p* < 0.001, Figure 7B). We could explain this result, in part, by the increased mortality, due to the low Ca^2+^ -conditions in the siOrai1 transfected cells, quantified by Trypan blue colorimetric assay (siCtrl 1.4 mM Ca^2+^: 5.97 ± 0.55%, siCtrl 0.1 mM Ca^2+^: 10.91 ± 1.93%, siOrai1 1.4 mM Ca^2+^: 7.93 ± 1.18%, siOrai1 0.1 mM Ca^2+^: 21.84 ± 1.83, N = 3, *p* < 0.001, Figure 7C). Moreover, TGF_β1_ secretion remained similar, between Orai1 knocked-down cells treated with low Ca^2+^-conditions and with physiological Ca^2+^ -conditions (siCtrl 1.4 mM Ca^2+^: 100 ± 8.77%, siCtrl 0.1 mM Ca^2+^: 67.79 ± 4.43%, siOrai1 1.4 mM Ca^2+^: 46.43 ± 8.91%, siOrai1 0.1 mM Ca^2+^: 49.66 ± 3.96%, N = 3, *p* > 0.05, Figure 7D).

Together, these data suggested that Ca^2+^ entry through Orai1 mediates AKT activation in order to regulate PS-1 cell proliferation and TGF_β1_ secretion.

## 3. Discussion

Since, Won et al. first characterized the Ca^2+^ signaling events present in PSCs in 2011, relatively few reports have continued to investigate the importance of Ca^2+^ and Ca^2+^ channels in PSC activation hallmarks [22]. While it is well known that PSC activation mediates pancreatic tumor’s desmoplastic reaction, this study is one of the first pieces of evidence showing the functional expression and role of the Orai1-Ca^2+^ channel in human PSCs’ activation processes. We demonstrated, for the first time, that Ca^2+^ entry through the Orai1 channel promotes activated PSC proliferation by controlling cell cycle progression in G0/G1 phase, as well as G1/S transition and TGF_β1_ secretion, via the activation of the AKT signaling pathway. Moreover, we found that TGF_β1_ enhances PSC proliferation via an autocrine positive feedback loop, which involves TGF_β1_ mediated Orai1-dependent AKT activation through the increase of Orai1 expression.

Whereas there is a very limited number of studies focusing on the importance of Orai1-CRAC channels in pancreatic cancer, we have previously demonstrated the functional expression of Orai1 in several pancreatic ductal adenocarcinoma cell lines [43]. However, the Orai1-CRAC channel has only recently been identified in PSCs [24,25]. CRAC channels were initially discovered in mouse quiescent PSCs by Gryshchenko et al., using the 2APB and GSK-7975A SOC pharmacological inhibitors, where they have shown that SOCs mediate bradykinin-elicited Ca^2+^ signals [24]. The same authors have then investigated the effect of GSK-7975A inhibitor in mouse activated PSCs in acute pancreatitis conditions, which seemed to prevent the increased responsiveness of PSCs to trypsin, known to be involved in PSC activation [24]. A year later, Waldron et al. have highlighted the presence of CRAC channels in mouse immortalized activated PSC cell line by demonstrating a marked expression of Orai1 and STIM1, the key actors of SOCs [25]. Our study strengthened these data by using a more specific approach leading to Orai1 protein silencing by siRNA transfection, showing Orai1 channel involvement in SOCE but also in the regulation of the Ca^2+^ basal concentration in human-activated PSCs. Nevertheless, there is no direct evidence of Orai1 function in PSC activation hallmarks so far.

Our results demonstrated an essential role of Orai1-mediated Ca^2+^ entry in the regulation of human-activated PSC proliferation, known to be involved in fibrotic desmoplasia development. The relationship between Ca^2+^ entry through the SOCs and cell proliferation has been demonstrated for a long time in many cell types, such as neural progenitor cells, osteoblasts, kidney cells, endothelial cells, and cancer cells, but not in PSCs [41,44,45,46,47]. Indeed, a decrease in SOCE amplitude is related to cell cycle arrest [48,49,50]. However, although the involvement of the Orai1 channel in cell proliferation is well established, most of the research evidence has shown that Orai1 mediates the G2/S phase of the cell cycle, especially in cancer cells [51,52,53,54]. Nevertheless, some other studies have proven that Orai1 can control G1/S transition in several cell types by downregulating the Cyclin E-CDK2 complex, known to be involved in this cell cycle phase transition, but not necessarily the G2/M transition [55]. These findings are in agreement with our data, showing that the knocking down of Orai1 in activated PSCs induced a cell number accumulation in G0/G1 phase and thus a decrease in G1/S transition, without affecting the G2/M phase.

Activated PSCs are also characterized by their ability to produce and secrete a variety of cytokines and growth factors, such as TGF_β1_, leading to the perpetuated activation of PSCs but also participating in the permanent dialogue with the pancreatic cancer cells [5]. Although the implication of Orai1-mediated Ca^2+^ entry has been well illustrated in immune cell’s cytokine production and secretion, some other studies showed the involvement of Orai1 in cytokine synthesis in bronchial cells, spinal astrocytes, and microglia [42,56,57]. However, the first study of Orai1-mediated Ca^2+^ entry involvement in fibro-inflammatory gene expression in activated PSCs emerged only in 2019. In this report, the researchers used the SOCE inhibitor CM4620 (≥10-fold selectivity for Orai1 vs. Orai2), providing novel data on SOCE mediated fibro-inflammatory gene expression, including TGF_β1_, within mouse activated PSCs, after lipopolysaccharide (LPS) stimulation [25]. This later is known to induce Orai1-mediated SOCE and cytokine production in mesenchymal cells. In agreement with these data, we demonstrated in the current work that Ca^2+^ entry through Orai1 mediates TGF_β1_ expression and secretion, the main profibrotic cytokine implicated in the desmoplastic reaction, in human-activated PSCs.

Furthermore, PSCs’ activation and cell functions are under the control of multiple signaling pathways and molecules, entraining dynamic cellular modifications [18]. One of the key intracellular pathways shown to be crucially involved in the regulation of PSC activation processes is the PI3K/AKT pathway [31]. Indeed, AKT is a serine-threonine kinase, initiator of PI3K cascade, described to play an important role in the development of pancreatic fibrosis by modulating PSC proliferation, migration, collagen production as well as growth factors and cytokine secretion [58,59,60]. Schwer et al. have reported that inhibition of the AKT pathway, using carbon monoxide releasing molecule-2, disrupted PSC activation by inducing a translational inhibition. This phenomenon caused inhibition of PSC proliferation due to the down-regulation of cyclin D1 and E protein expression and the interruption of G1/S cell phase progression [20]. In addition, Zhang et al. have shown that reduction in AKT activity by the tumor suppressor PTEN led to cyclin D1 down-regulation and inhibition of cell proliferation in human and rodent activated PSCs [21].

Moreover, according to the literature, AKT pathway activation requires Ca^2+^ influx. It has been demonstrated that Ca^2+^ entry through Orai1 induces AKT phosphorylation in order to stimulate cell proliferation in several cell types, such as esophageal squamous cancer cells, colorectal and non-small lung cancer cells [46,61,62]. Furthermore, it has recently been established that Orai1 inhibition, using the RP4010 CRAC channel inhibitor, decreased pancreatic cancer cell proliferation through the down-regulation of the AKT/mTOR signaling pathway [63]. However, we were the first to illustrate activation of the AKT pathway via Orai1-mediated Ca^2+^ entry resulting in cell proliferation enhancement in human-activated PSCs. In our study, on the one hand, Orai1 silencing decreased AKT phosphorylation without affecting ERK1/2 and SMAD2 activation, which are two of the main pathways modulating PSC activation. On the other hand, low extracellular Ca^2+^ concentration led to an inhibition of AKT phosphorylation and PSC proliferation, suggesting that Orai1-mediated Ca^2+^ entry is indispensable for AKT activation in order to stimulate PSC proliferation. This data, on the primordial role of the AKT pathway in the regulation of PSC proliferation, was also confirmed using the known PI3K/AKT pharmacological inhibitor LY294002.

However, the high mitotic index of activated PSCs alone is insufficient to induce pancreatic fibrogenesis during chronic pancreatitis and pancreatic cancer, leading to dense fibrotic desmoplasia development. To date, the most potent fibrogenic factor involved in PSC-mediated fibrosis, as well as in the interaction with the pancreatic cancer cells, is the TGF_β1_. This pleiotropic cytokine promotes PSC activation by regulating α-SMA expression, type I collagen synthesis, and cell proliferation in an autocrine manner. Indeed, in turn, activated PSCs trigger TGF_β1_ auto-synthesis to maintain their activation. Ohnishi et al. have reported that TGF_β1_ expression and secretion are mediated through the activation of the ERK1/2 pathway in rat-activated PSCs [32,64]. In contrast, TGF_β1_ mRNA and protein expression are regulated by the PI3K/AKT pathway in human-activated hepatic stellate cells, counterparts of PSCs, which is in line with our findings in human-activated PSCs [34]. We have shown that PSC treatment with LY294002 significantly decreases TGF_β1_ mRNA expression and secretion, suggesting that TGF_β1_ synthesis and secretion in human-activated PSCs is AKT-dependent. Interspecies differences could explain TGF_β1_ synthesis and secretion-dependent pathway discordance between rat and human-activated PSCs. Besides AKT-dependent TGF_β1_ secretion, we have demonstrated that Ca^2+^ is essential for this process since extracellular Ca^2+^ reduction led to decreased-TGF_β1_ secretion. In addition, we proposed in this study a mechanism by which Ca^2+^ influx through Orai1 regulates TGF_β1_ secretion via an AKT-dependent pathway. When we inhibited Orai1 expression under low extracellular Ca^2+^ concentrations, we did not observe any additional effect on TGF_β1_ secretion compared to Orai1 knocked-down cells under physiological extracellular Ca^2+^ concentrations. These findings suggested that Ca^2+^ entry through Orai1 induces AKT phosphorylation to promote TGF_β1_ secretion in human-activated PSCs.

The most interesting finding in this report is the identification of a TGF_β1_-mediated autocrine positive feedback loop, promoting PSC Orai1/AKT-dependent proliferation through Orai1 activity and expression increase, leading to a perpetuated PSC activation. A link between TGF_β1_ and Orai1 has previously been reported in airway smooth muscle cells, showing a SOCE stimulation due to Orai1 mRNA expression increase, promoted by TGF_β1_ long-term exposure [65]. Following these findings, we were the first to illustrate that TGF_β1_ treatment induced a rise of Orai1 mRNA and protein amounts in human-activated PSCs, leading to Orai1-mediated Ca^2+^ entry promotion. Indeed, the TGF_β1_ effect on Orai1 activity was observed only by TGF_β1_ perfusion in our cellular model. Furthermore, several studies have demonstrated a stimulatory role of TGF_β1_ on Ca^2+^ entry in a context-dependent manner. For example, TGF_β1_ long- but not short-term treatment has been shown to increase voltage-operated channel-mediated Ca^2+^ entry in hepatic stellate cells [66], whereas TGF_β1_ short treatment was sufficient to promote Ca^2+^ influx through TRPC6 in intestinal myofibroblasts [40]. Additionally, TGF_β1_ perfusion raised Ca^2+^ release from the endoplasmic reticulum and so the SOCE in pancreatic cancer cells, suggesting its crucial involvement in pancreatic cancer as well [38]. Furthermore, we have provided evidence that TGF_β1_-mediated Orai1-Ca^2+^ entry and expression stimulate Orai1/AKT activation and proliferation. Cell treatment with exogenous TGF_β1_ increased AKT phosphorylation in siCtrl transfected cells, while this rise of AKT activity was partially absent in Orai1-knocked-down cells under TGF_β1_ treatment. It has already been established by Tsang et al. that TGF_β1_ can activate the AKT pathway by increasing its phosphorylation in rat-activated PSCs [67], and these data were confirmed here in human-activated PSCs. Moreover, we have reported a role of TGF_β1_ in the promotion of PSC proliferation through Orai1-mediated AKT activation. Indeed, PSC proliferation increased after TGF_β1_ treatment compared to non-treated cells, while Orai1 silencing abolished the stimulatory effect of TGF_β1_ on PSC proliferation. Unlike our results, most of the studies conducted on PSCs have shown an inhibitory effect of TGF_β1_ on PSC proliferation. These reports have been realized on rat-activated PSCs, demonstrating that TGF_β1_ inhibits PSC proliferation through a Smad3-dependent pathway and by an accumulation of the G1 phase inhibitors p21 and p27, inducing G0/G1 cell cycle arrest [68]. TGF_β1_ has been shown to induce both growth promotion and inhibition within the same cell type, depending on the context but also on the abundance or activity of extracellular TGF_β1_ ligands, which could partly explain TGF_β1_ promoted proliferation in our human-activated PSCs [13,69,70]. Elsner et al. have reported an indirect stimulatory effect of TGF_β1_ on rat-activated PSC proliferation through the AKT pathway, using the multi-kinase inhibitor sorafenib [71]. TGF_β1_ has been shown to regulate cell growth through a Smad-dependent and Smad-independent pathway. The latter consisted of AKT activation [17]. Indeed, it has been reported that TGF_β1_-induced AKT phosphorylation inhibits Smad3-mediated growth inhibition by binding to and sequestering Smad3 in the cytosol, leading to growth promotion, a mechanism that could justify our present results [72,73].

## 4. Conclusions

**Figure 8:**
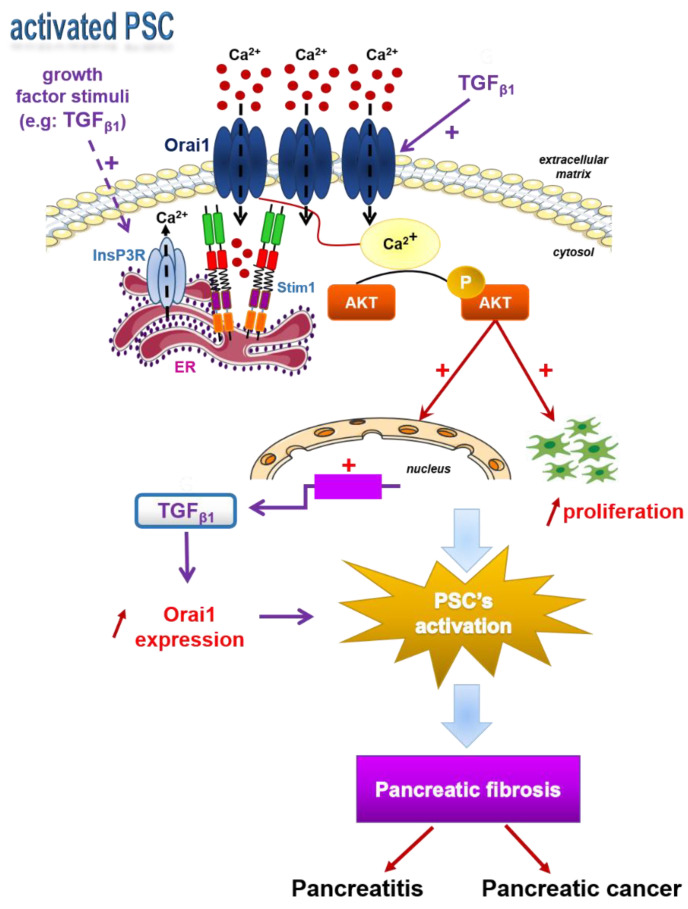
Conclusive scheme: Endoplasmic reticulum Ca^2+^ store depletion activates Orai1 channel permitting SOCE. Ca^2+^ entry through Orai1 activates AKT phosphorylation, which stimulates human-activated PSC proliferation and TGF_β__1_ expression and secretion, enhancing PSC activation to promote pancreatic fibrosis, and so pancreatitis and pancreatic cancer. In turn, TGF_β__1_ secretion stimulates Orai1-mediated Ca^2+^ entry by simultaneously increasing Orai1 mRNA and protein expression in order to stimulate Orai1/AKT-dependent proliferation, maintaining PSC activation.

Taken together, this is the first study revealing the important role of the Orai1 channel in PSC physiology, suggesting a role in fibrotic desmoplasia development, the main feature of PDAC. We suggest in this report that Orai1 can be involved in maintaining PSC activation to perpetuate pancreatic fibrosis by promoting PSC proliferation and TGF_β1_ expression and secretion through an AKT-dependent pathway. More importantly, we show that secreted TGF_β1_ from activated PSCs induces an autocrine positive feedback loop by stimulating Orai1-mediated Ca^2+^ entry and increasing Orai1 expression. This later results in the promotion of Orai1-dependent AKT phosphorylation and thus to PSC proliferation enhancement to exacerbate pancreatic fibrosis development (Figure 8). Therefore, compounds that inhibit PSC proliferation by targeting Orai1 activity directly or the downstream effectors by inhibiting PI3K/AKT pathway or TGF_β1_ synthesis and/or secretion may have the potential to become a new approach for PDAC treatment or limit the drug resistance.

## 5. Materials and Methods

### 5.1. Cell Culture

The PS-1 pancreatic human stellate cell line was generously given by Pr Hemant M. Kocher from the Queen Mary University of London. PS-1 cells were isolated from a healthy donated human pancreas and immortalized with retroviruses containing cDNA encoding human telomerase reverse transcriptase (hTERT) and selected with puromycin, as previously described [74]. RLT PSCs were established from a chronic pancreatic tissue resection and immortalized with SV40 large T antigen and the catalytic subunit of hTERT, as previously described [75]. PS-1 and RLT PSCs were grown in Dulbecco’s Modified Eagle Medium/Nutrient Mixture F-12 (DMEM/F12, Gibco, Thermo Fischer Scientific, Illkirch, France) supplemented with 10% Fetal Bovine Serum (FBS, Pan Biotech, Dominique Dutscher, Brumath, France). Cells were maintained at 37 °C in a humidified atmosphere containing 5% CO_2,_ and the cell culture medium was changed every 48 h.

### 5.2. Cell Transfection and RNA Interference

Cells were transfected with small interfering RNA (siRNA) by electroporation, using the nucleofection technology (Amaxa Biosystems, Lonza, Aubergenville, France). PS-1 and RLT cells (10^6^) were transiently nucleofected according to the manufacturer’s protocol with 4 µg of scrambled siRNA as a control (siCtrl: duplex negative control, Eurogentec) or with siRNA directed against Orai1 (siOrai1: 5′-CGUGCACAAUCUCAACUCG-3′, Eurogentec). All the experiments were performed 72 h after the siRNA transfection.

### 5.3. Chemicals and Reagents

Pharmacological inhibitors and cytokines used to study the signaling pathways were the following: LY-294002 (20 µM, 72 h, Sigma-Aldrich, St. Quentin Fallavier, France), and transforming growth factor-_β1_ human (20 ng/mL for 48 h treatment and 30 min stimulation, 5 ng/mL for perfusion, Sigma-Aldrich). The chemical ethylene glycol-bis (β-aminoethyl ether)-*N,N,N’,N’*-tetraacetic acid (EGTA, 1.3 mM, 72 h treatment and 30 min stimulation, Sigma-Aldrich) was used to chelate extracellular Ca^2+^. The optimal EGTA concentration in order to reach 0.1 mM of extracellular Ca^2+^ was determined using the max-chelator software (https://somapp.ucdmc.ucdavis.edu/pharmacology/bers/maxchelator/CaMgATPEGTA-TS.htm, accessed on 10 October 2019)

### 5.4. Cell Proliferation Assay

PS-1 and RLT cells were seeded in 6-well plates (8 × 10^4^ cells per well for PS-1, 6 × 10^4^ cells per well for RLT), and cell proliferation was assessed 72 h after siRNA transfection or treatment with pharmacological inhibitors or TGF_β1_ cytokine, as described above, by MTT assay. Cells were incubated with the 3-(4,5-dimethylthiazol-2-yl)-2,5-diphenyltetrazolium bromide (MTT, Sigma-Aldrich, Inc.) solubilized in culture medium (0.5 mg/mL), for 45 min at 37 °C in the dark, to be converted to an insoluble formazan. To dissolve the formazan crystals, the culture medium was replaced by dimethyl sulfoxide (DMSO, Sigma-Aldrich, Inc.), and the absorbance was measured at 550 nm using an Infnite^®^ 200 Pro reader (Tecan Trading AG, Männedorf, Switzerland).

### 5.5. Cell Cycle Analysis

DNA cellular content quantitation by flow cytometry was used for cell cycle evaluation. Cells transfected (1 × 10^6^) with siOrai1 or siCtrl, were firstly fixed with cold absolute ethanol (≥99.8%, Sigma-Aldrich) for at least 6 h at 4 °C. Then, cells were pelleted, resuspended in PBS-5 mM EDTA, treated with 20 mg/mL RNAseA (Sigma-Aldrich) for 30 min at ambient temperature, and stained with 50 mg/mL of propidium iodide (Sigma-Aldrich, St. Quentin Fallavier, France). Samples were then analyzed by flow cytometer (Accuri^®^, Dominique Dutscher, Brumath, France), and the cell percentage in different phases was calculated using Cyflogic software. 

### 5.6. Cell Mortality

PS-1 cells were grown in 35 mm Petri-dishes at a density of 8 × 10^4^ cells, and RLT cells at a density of 6 × 10^4^, for 72 h, after siRNA transfection, and then cell death was measured by trypan blue assay. Cells were removed by trypsinization, diluted in trypan blue solution (Sigma-Aldrich), and counted six times using the standard Malassez cell method. The number of cell mortality was obtained using the formula: rate of cell death = number of dead cells/number of total cells, normalized to control. This colorimetric assay also provided us the number of cell proliferation, obtained using the formula: rate of cell proliferation = number of alive cells * 4 * 1900, normalized to control.

### 5.7. Apoptosis Analysis

The apoptosis process was assessed by studying the cell surface of phosphatidylserine exposure on the outer leaflet of the plasma membrane, an early marker of apoptotic cell death. Both detached and adherent cells were collected, washed twice in ice-cold PBS, and resuspended in 1× binding buffer (BD Biosciences Pharmingen, Le Pont de Claix, France). Apoptotic cells were determined using a PE Annexin V Apoptosis Detection Kit staining (BD Biosciences Pharmingen), which consisted of adding FITC Annexin V and propidium iodide (PI) to the cell preparations and incubating them for 15 min at 25 °C in the dark. Binding buffer was then added to each tube, and the samples were analyzed by a flow cytometer (Accuri^®^). Compensation and quadrants were set up using the following controls: unstained cells, cells stained only with FITC Annexin V, and cells stained only with PI. 

### 5.8. Calcium Imaging

Store-operated calcium entry (SOCE) was measured by calcium imaging using the ratiometric probe Fura-2/AM. Transfected cells (8 × 10^4^ for PS-1 and 6 × 10^4^ for RLT) were plated on glass coverslips in 35 mm Petri-dishes and loaded with 3 µM Fura-2/AM (Sigma-Aldrich) in extracellular saline solution for 45 min at 37 °C before Ca^2+^ measurement. After Fura-2 incubation, cells were washed three times and kept in the extracellular saline solution containing 145 mM NaCl, 5 mM KCl, 10 mM HEPES, 5 mM glucose, 2 mM CaCl_2_, and 1 Mm MgCl_2_, at pH 7.4. The coverslip was transferred onto a perfusion chamber on a Zeiss microscope equipped for fluorescence. Fura-2 fluorescence was excited alternatively at 340 and 380 nm using a monochromator (polychrome IV, TILL Photonics, Planegg, Germany) and captured by a Cool SNAPHQ camera (Princeton Instruments, Evry, France) after filtration through a long-pass filter (510 nm emission wavelength). Signal acquisition and analysis were obtained with Metafluor software (version 7.1.7.0, Molecular Devices, St. Grégoire, France). The intracellular Ca^2+^ concentration was derived from the ratio of the fluorescence intensities for each of the excitation wavelengths (F_340_/F_380_). Cells were continuously perfused with the saline solution, and all recordings were performed at room temperature. SOCE was induced after 1 µM Thapsigargin (Sigma-Aldrich) stimulation in Ca^2+^-free solution for 11 min, followed by 2 mM Ca^2+^ perfusion for 10 min. The flow rate of the whole-cell chamber perfusion system was set to 1 mL/min, and the chamber volume was 700 µL. 

### 5.9. qRT-PCR

Total cellular RNA was extracted by the standard Trizol reagent (Sigma-Aldrich) method, and RNA concentration and purity were determined by using a spectrophotometer (NanoDrop 2000, Wilmington, NC, USA). Then, cDNA was synthesized with a MultiScribe™ Reverse Transcriptase kit (Applied Biosystems, Carlsbad, CA, USA) from 2 µg of RNA. For the real-time PCR, sense, and antisense PCR primers specific to Orai1 (forward 5′-AGGTGATGAGCCTCAACGAG-3′ and reverse 5′-CTGATCATGAGCGCAAACAG-3′), TGF_β1_ (forward 5′-ACATCAACGCAGGGTTCACT-3′ and reverse 5′-GAAGTTGGCATGGTAGCCCT-3′), and HPRT1 (forward 5′-AGTTCTGTGGCCATCTGCTT-3′ and reverse 5′-CAATCCGCCCAAAGGGAACT-3′) were used. Real-time PCR was performed on a LightCycler System (Roche, Basel, Switzerland) using LightCycler 480 SYBR Green I PCR master mix (Life Science, Roche). Orai1 and TGF_β1_ mRNA expression were normalized to the endogenous gene control (HPRT1) and compared to the siCtrl sample, using the Pfaffl method [76].

### 5.10. TGF_β1_ Dosage Assay

Enzyme-Linked Immunosorbent Assay kit for TGF_β1_ quantitative measurement from cell culture supernatant was realized according to the technical protocol provided by the supplier (Sigma-Aldrich). All samples dosed for TGF_β1_ were treated 10 min with 1 N HCl at room temperature to activate latent TGF_β1_ to the immunoreactive form and then neutralized with 1.2 N NaOH/0.5 N HEPES. The absorbance from the colorimetric reaction corresponding to the TGF_β1_ quantity contained in the supernatant was read at 450 nm.

### 5.11. Western Blotting and Co-Immunoprecipitation

Cells were lysed in RIPA buffer (1% Triton X-100, 0.1% sodium deoxycholate, 150 mM NaCl, 10 mM PO4Na2/K, pH = 7.4) containing protease inhibitor cocktail (Sigma-Aldrich), 5 mM sodium orthovanadate and 2 mM EDTA. Protein concentration was determined by the Bicinchoninic Acid protein assay (Bio-Rad, Marnes-La-Coquette, France). For standard Western blotting, 30 µg of denatured protein lysate from each sample was loaded in SDS-PAGE, separated by the denaturing SDS-PAGE, and transferred onto a nitrocellulose membrane (Dominique Dutscher, Brumath, France). The primary antibodies used were: anti-Orai1 (1:250, Sigma-Aldrich), anti-αSMA (1:1000, Abcam), anti-GAPDH (1:4000, Abcam), anti-SMAD2 (1:1000, Abcam), anti-p-SMAD2 (phospho S255) (1:1000, Abcam), anti-ERK1/2 (1:500, Cell Signaling), anti-p-ERK1/2 (Thr202/Tyr204) (1:500, Cell Signaling), anti-Akt (1:500, Cell Signaling) and anti-p-Akt (Ser473) (1:500, Cell Signaling). The secondary antibodies used were coupled to horseradish peroxidase, which permitted protein band detection through an enhanced chemiluminescence kit (Ozyme). Protein bands were quantified using the densitometric analysis option in the Bio-Rad image acquisition software (Quantity One), and all experiments results were normalized to the GAPDH, used as a control referent protein.

Co-immunoprecipitation experiments were realized with 500 µg of protein lysates, precleared for 1 h 30 with proteins A sepharose magnetic beads (Millipore, PureProteome™) and then incubated overnight with the primary antibody. The dilution of primary antibodies used for the co-immunoprecipitation experiments was anti-Orai1 1:100 (Sigma-Aldrich) and anti- αSMA 1:200 (Abcam). Then, the antigen-antibody complex was precipitated with protein A sepharose magnetic beads (Millipore, PureProteome™) for 1 h. After denaturation, proteins were used for a standard Western blot, as described above.

### 5.12. Statistical Analysis

All data are presented as mean ± SEM (standard error of the mean), n corresponds to the number of cells, and N refers to the number of cell passages. All experiments were performed at least with three different cell passages. Statistically significant differences were determined with paired or unpaired *t*-test or with one-way or two-way ANOVA and post hoc Bonferroni test for multiple comparisons, depending on the compared conditions, using GraphPad Prism version 5 (GraphPad Software, La Jolla, CA, USA). Differences between the values were considered significant when *p* < 0.05. The *p*-values < 0.05, <0.01, and <0.001 are represented as *, **, and ***, respectively.

## Data Availability

Data are contained within the article or supplementary material.

## Supplementary Materials

The following are available online at https://www.mdpi.com/article/10.3390/cancers13102395/s1, Figure S1: (A) Localization of Orai1 channel in PS-1 visualized by confocal microscopy, 72h post-proliferation. (B) Orai1 channel mediates the Ca2+ basal concentration in both, PS-1 and RLT human activated PSC cell lines. (C) SOCs channels are opened in cell culture conditions due to the permanent ER-Ca2+ depletion induced by FBS. Figure S3: Orai1 knocking-down impacts SMAD2 total protein expression without affecting AKT and ERK1/2 in PS-1 human activated PSCs, Figure S4: Orai1 modulates AKT activation but not ERK1/2 nor SMAD2 phosphorylation in RLT human activated PSCs, without affecting their total protein amount. Figure S5: Orai1 channel neither regulates αSMA expression nor colocalizes with αSMA in human activated PSCs.
