# Supplementary material for: Orai1 Channel Regulates Human-Activated Pancreatic Stellate Cell Proliferation and TGFβ1 Secretion through the AKT Signaling Pathway
Source: Cancers (Basel). 2021 May 15;13(10):2395. doi: 10.3390/cancers13102395 (PMC8156432; doi:10.3390/cancers13102395)
Supplement: Supplementary file 1 [file cancers-13-02395-s001.zip › cancers-1168600-supplementary.pdf]

cancers

MDPI

Article

Orai1 Channel Regulates Human-Activated Pancreatic Stellate Cell Proliferation and TGFβ1 Secretion through the AKT Signaling Pathway

Silviya Radoslavova, Antoine Folcher, Thibaut Lefebvre, Kateryna Kondratska, Stéphanie Guénin, Isabelle Dhennin-Duthille, Mathieu Gautier, Natalia Prevarskaya and Halima Ouadid-Ahidouch

Supplementary Figures

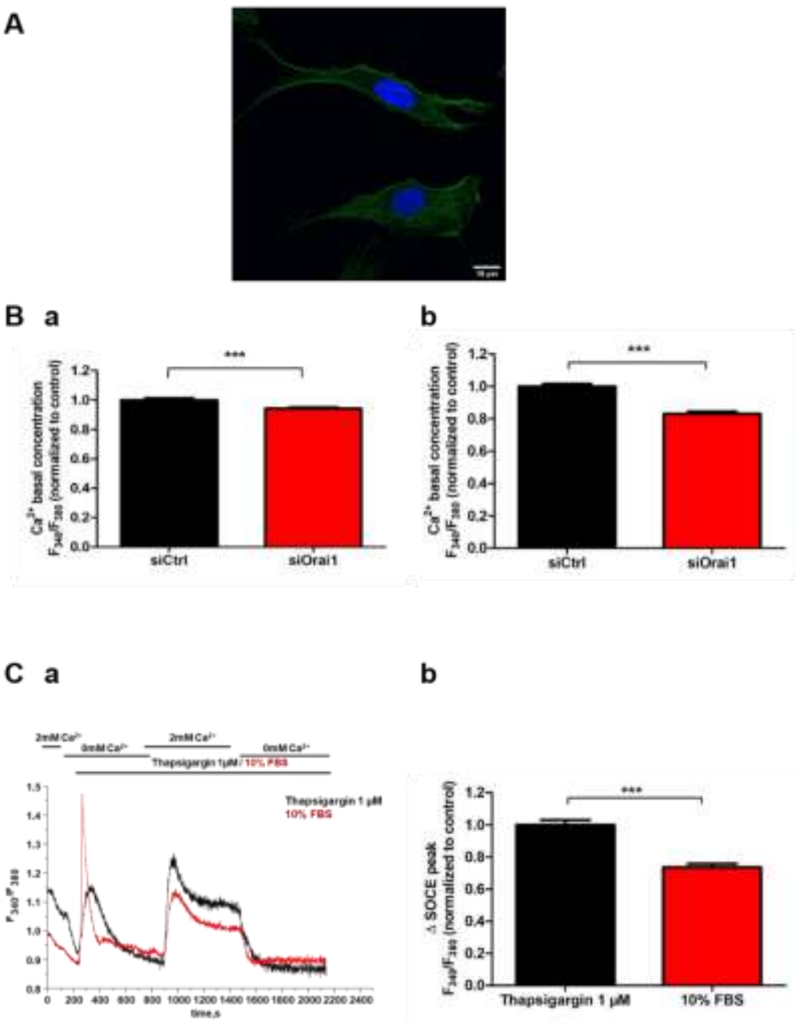

Figure S1. (**A**) Localization of Orai1 channel in PS-1 visualized by confocal microscopy, 72h post-proliferation. (**B**) Orai1 channel mediates the Ca^{2+} basal concentration in both, PS-1 and RLT human activated PSC cell lines. Quantification of Ca^{2+} basal concentration in PS-1 cells (**B-a**) (siCtrl: n = 210, siOrai1: n = 189, N = 5) and RLT cells (**B-b**) (siCtrl: n = 92, siOrai1: n = 80, N = 3), after 72h of Orai1 inhibition. Histograms are represented as the average ± SEM normalized to the control. (**C**) SOCs channels are opened in cell culture conditions due to the permanent ER-Ca^{2+} depletion induced by FBS. Illustration of representatives SOCE measurement traces, after perfusion of 1 µM Thapsigargin and 10% FBS, in PS-1 no-treated cells, 72h post-proliferation (**C-a**). Cells were starved overnight, the day before the experiments. SOCE

quantification was normalized to the control condition (Thapsigargin) (**C-b**). FBS perfusion induced a lower SOCE than Thapsigargin perfusion (Thapsigargin: n = 87, FBS: n = 78, N = 3). All values were reported as mean ± SEM. (*** $p < 0.001$, Student's *t*-test, n: number of cells, N: number of passage).

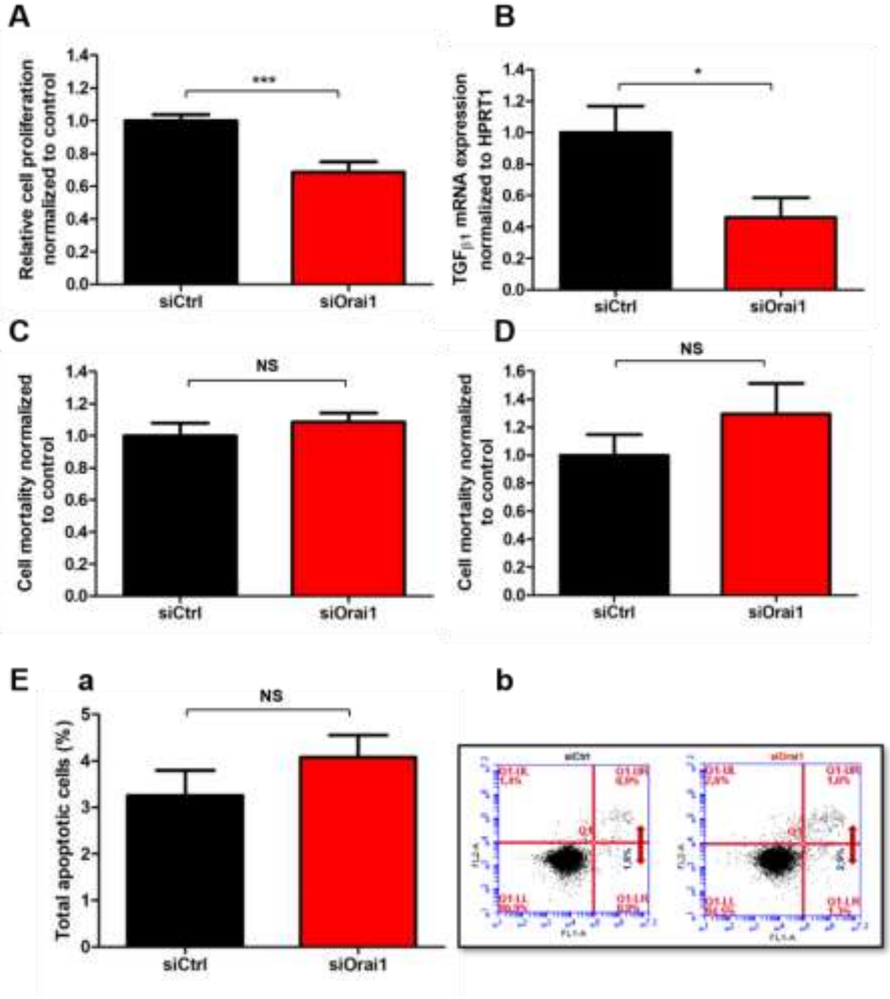

Figure S2. Orai1 is involved in the regulation of RLT-PSC's proliferation and $TGF_{\beta1}$ expression without affecting the human activated PSC's survival. (**A**) Effect of Orai1 inhibition on RLT cell proliferation, 72 h post-transfection, evaluated by MTT assay. (**B**) Involvement of Orai1 in the modulation of $TGF_{\beta1}$ mRNA expression in RLT cells, assessed by qPCR, after 72 h of siOrai1 transfection. (**C,D**) Impact of Orai1 inhibition on PS-1 and RLT cell mortality, evaluated by Trypan Blue assay, 72h post-transfection. (**E**) Measurement of total apoptosis rate using annexin V staining in PS-1 siOrai1 transfected cells (**E-a**), with a representative apoptosis profile after 72h of Orai1 silencing (**E-b**). Values were normalized to control and reported as mean ± SEM, each experiment was performed at least in triplicate. (*** $p < 0.001$, * $p < 0.05$, NS, N = 3, Student *t*-test).

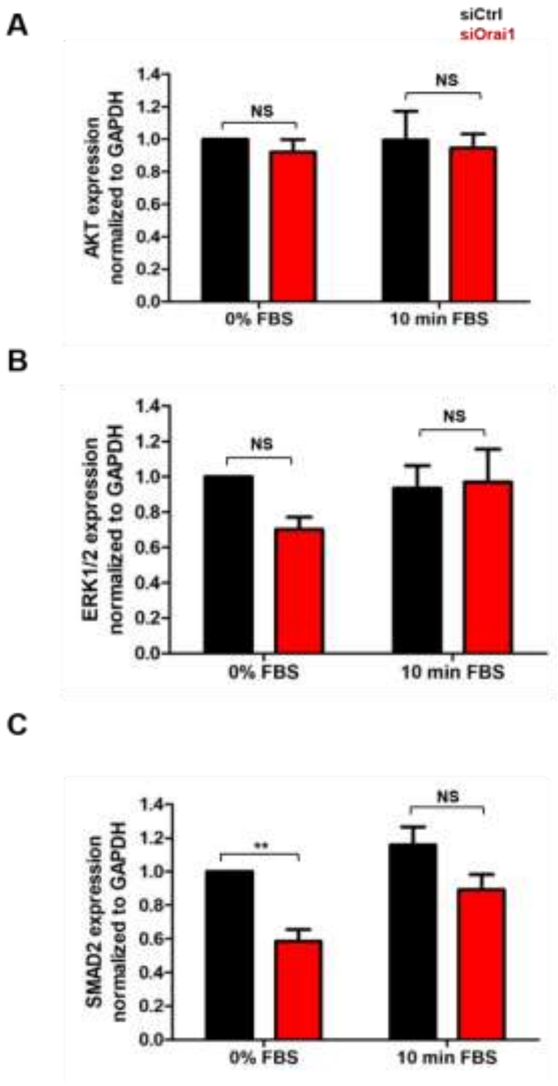

Figure S3. Orai1 knocking-down impacts SMAD2 total protein expression without affecting AKT and ERK1/2 in PS-1 human activated PSCs. Quantification of AKT (**A**), ERK1/2 (**B**), and SMAD2 (**C**) total protein expression in siOrai1 transfected cells. All values were first normalized to the referent protein GAPDH and then to the 0% FBS control condition. All experiments were performed 72 h post-transfection. Values were reported as mean ± SEM (** $p < 0.01$, NS, at least N = 3 two-way ANOVA followed by Bonferroni *post hoc* test).

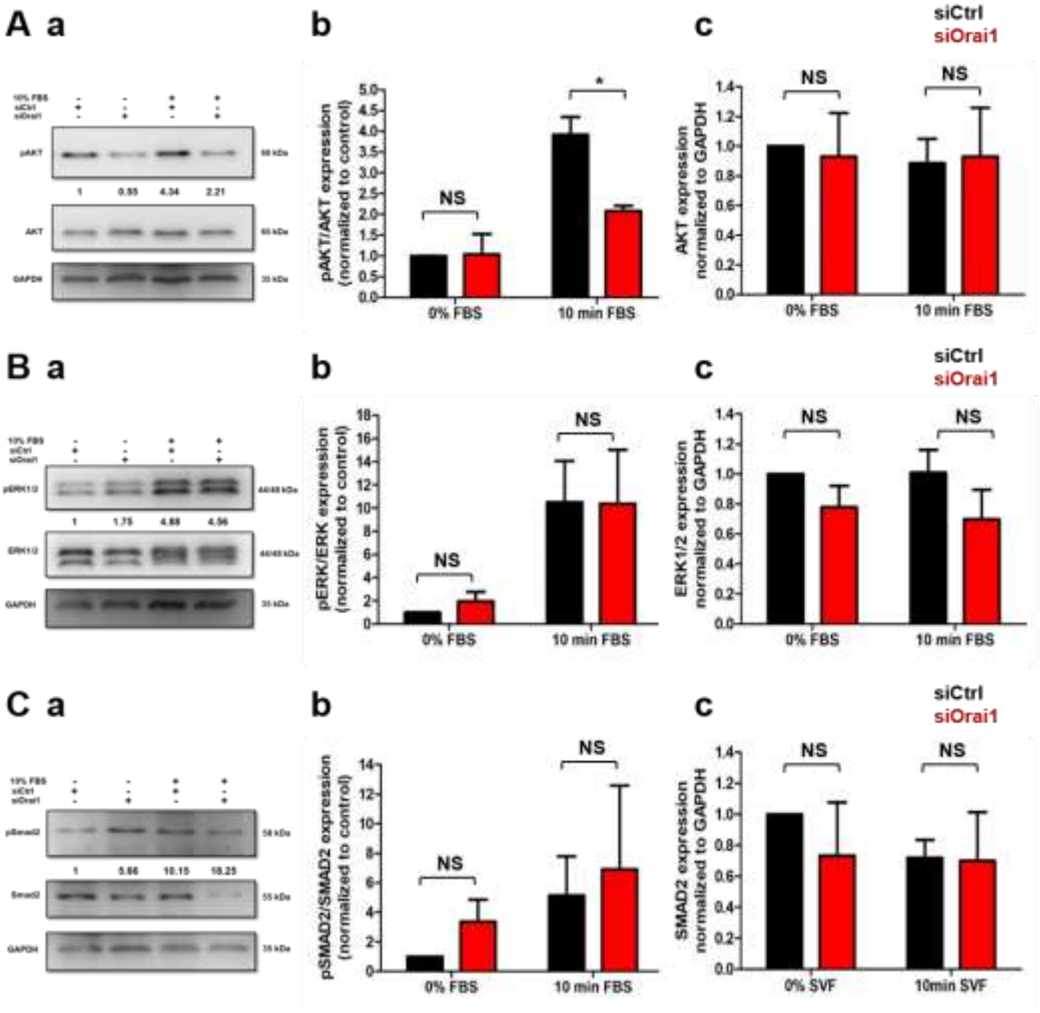

Figure S4. Orai1 modulates AKT activation but not ERK1/2 nor SMAD2 phosphorylation in RLT human activated PSCs, without affecting their total protein amount. (**A**) Implication of Orai1 in AKT phosphorylation in RLT cells. Representative Western blot showing the effect of Orai1 inhibition after FBS starvation of transfected cells overnight (**A-a**). Cells were then stimulated 10 min with FBS to evaluate the impact of Orai1 on AKT activation. AKT phosphorylation was quantified by the ratio of phosphorylated AKT form/total AKT protein (siCtrl+10 min FBS: 3.92 ± 0.43-fold, siOrai1+10 min FBS: 2.09 ± 0.13 fold, (**A-b**) and in parallel, the effect on the total protein expression was measured (**A-c**). (**B**) Assessment of ERK1/2 activation and total protein amount after Orai1 knocking down in RLT cells. Representative Western blot showing the effect of Orai1 silencing on ERK1/2 activation, using the protocol described above (**B-a**). ERK1/2 phosphorylation was quantified by the ratio of phosphorylated ERK1/2 form/total ERK1/2 protein (**B-b**), as well as the total protein expression (**B-c**). (**C**) Evaluation of Orai1 silencing on SMAD2 phosphorylation (**C-b**) and SMAD2 total protein expression (**C-c**). Representative Western blot showing the effect of siOrai1 transfected cells on SMAD2 activation (**C-a**), with the quantification using the ratio of phosphorylated SMAD2 form/total SMAD2 protein (**C-b**). All values were first normalized to the referent protein GAPDH and then to the 0% FBS control condition. All experiments were realized 72 h post-transfection. Values were reported as ± SEM (* $p < 0.05$, NS, N = 3, two-way ANOVA followed by Bonferroni *post hoc* test).

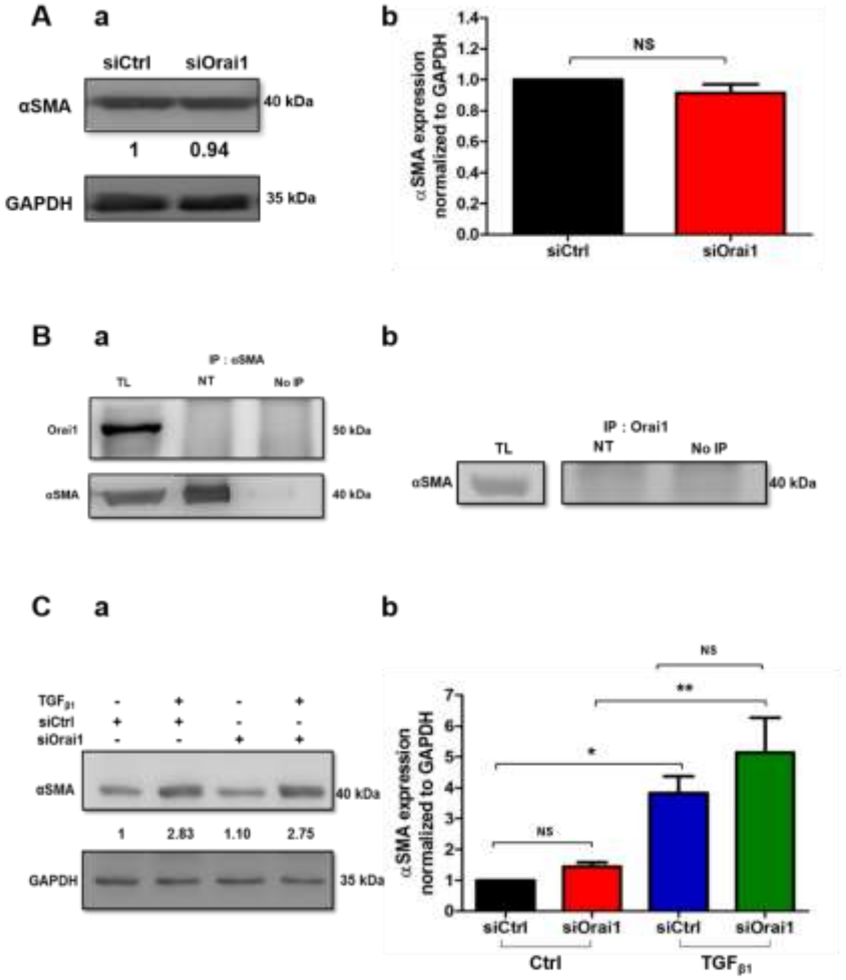

Figure S5. Orai1 channel neither regulates αSMA expression nor colocalizes with αSMA in human activated PSCs. (**A**) Orai1 silencing did not affect αSMA protein expression. Representative Western blot showing the impact of 72h Orai1 inhibition on αSMA expression (**A-a**) and the quantification (**A-b**) (NS, N = 3, Student *t*-test). The values were first normalized to the referent protein GAPDH and then to the control condition, reported as mean ± SEM. (**B**) Representatives Western blot of Orai1 and αSMA expression after immunoprecipitation experiments using anti-αSMA (**B-a**) and anti-Orai1 antibodies (**B-b**) (N = 3), showing the absence of physical interaction between the two proteins. (**C**) Effect of 72 h Orai1 knocked-down cells, treated 48h with $TGF_{\beta 1}$ (20 ng/mL), in the presence of low-FBS conditions (1%), on αSMA expression. Representative Western blot of αSMA expression (**a**) and the quantification (**b**) (* $p < 0.05$, ** $p < 0.01$, NS, N = 4, one-way ANOVA followed by Bonferroni multiple comparison test). The values were first normalized to the referent protein GAPDH and then to the control condition, reported as mean ± SEM.
